# The Mysterious Multitude: Structural Perspective on the Accessory Subunits of Respiratory Complex I

**DOI:** 10.3389/fmolb.2021.798353

**Published:** 2022-01-03

**Authors:** Abhilash Padavannil, Maria G. Ayala-Hernandez, Eimy A. Castellanos-Silva, James A. Letts

**Affiliations:** Department of Molecular and Cellular Biology, University of California, Davis, Davis, CA, United States

**Keywords:** mitochondrial complex I, oxidative phosphorylation (OXPHOS), accessory subunits, mitochondrial diseases, electron transport chain

## Abstract

Complex I (CI) is the largest protein complex in the mitochondrial oxidative phosphorylation electron transport chain of the inner mitochondrial membrane and plays a key role in the transport of electrons from reduced substrates to molecular oxygen. CI is composed of 14 core subunits that are conserved across species and an increasing number of accessory subunits from bacteria to mammals. The fact that adding accessory subunits incurs costs of protein production and import suggests that these subunits play important physiological roles. Accordingly, knockout studies have demonstrated that accessory subunits are essential for CI assembly and function. Furthermore, clinical studies have shown that amino acid substitutions in accessory subunits lead to several debilitating and fatal CI deficiencies. Nevertheless, the specific roles of CI’s accessory subunits have remained mysterious. In this review, we explore the possible roles of each of mammalian CI’s 31 accessory subunits by integrating recent high-resolution CI structures with knockout, assembly, and clinical studies. Thus, we develop a framework of experimentally testable hypotheses for the function of the accessory subunits. We believe that this framework will provide inroads towards the complete understanding of mitochondrial CI physiology and help to develop strategies for the treatment of CI deficiencies.

## Introduction

**FIGURE 1:**
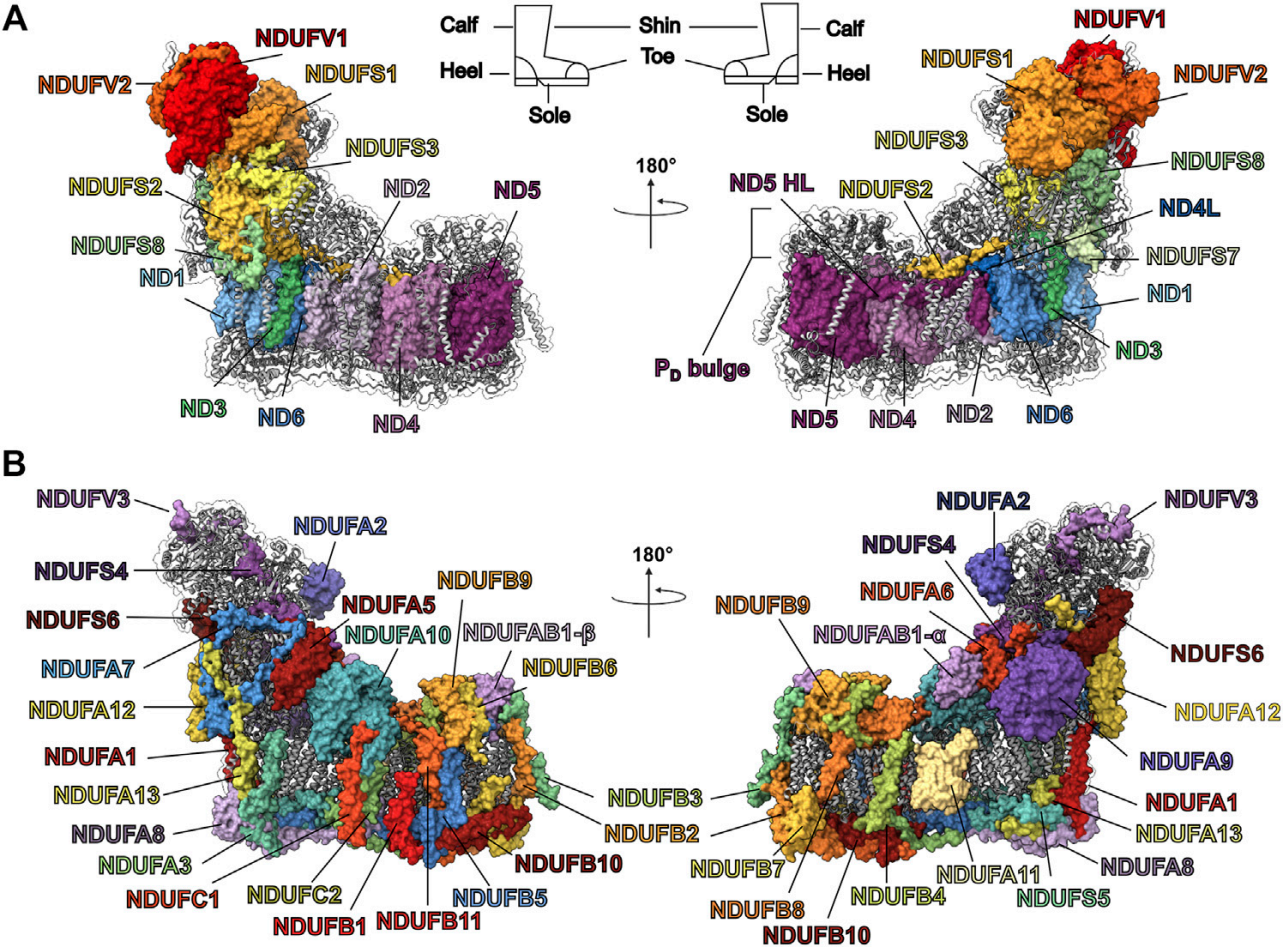
Structure of mammalian CI from *O. aries* (PDB: 6ZKC). **(A)** The 14 core subunits are shown as colored surfaces. The accessory subunits are shown as grey cartoons. The CI boot-shape analogy is indicated at the top of the panel. **(B)** The 31 accessory subunits are shown as colored surfaces while the core subunits are shown as grey cartoons.

**FIGURE 2:**
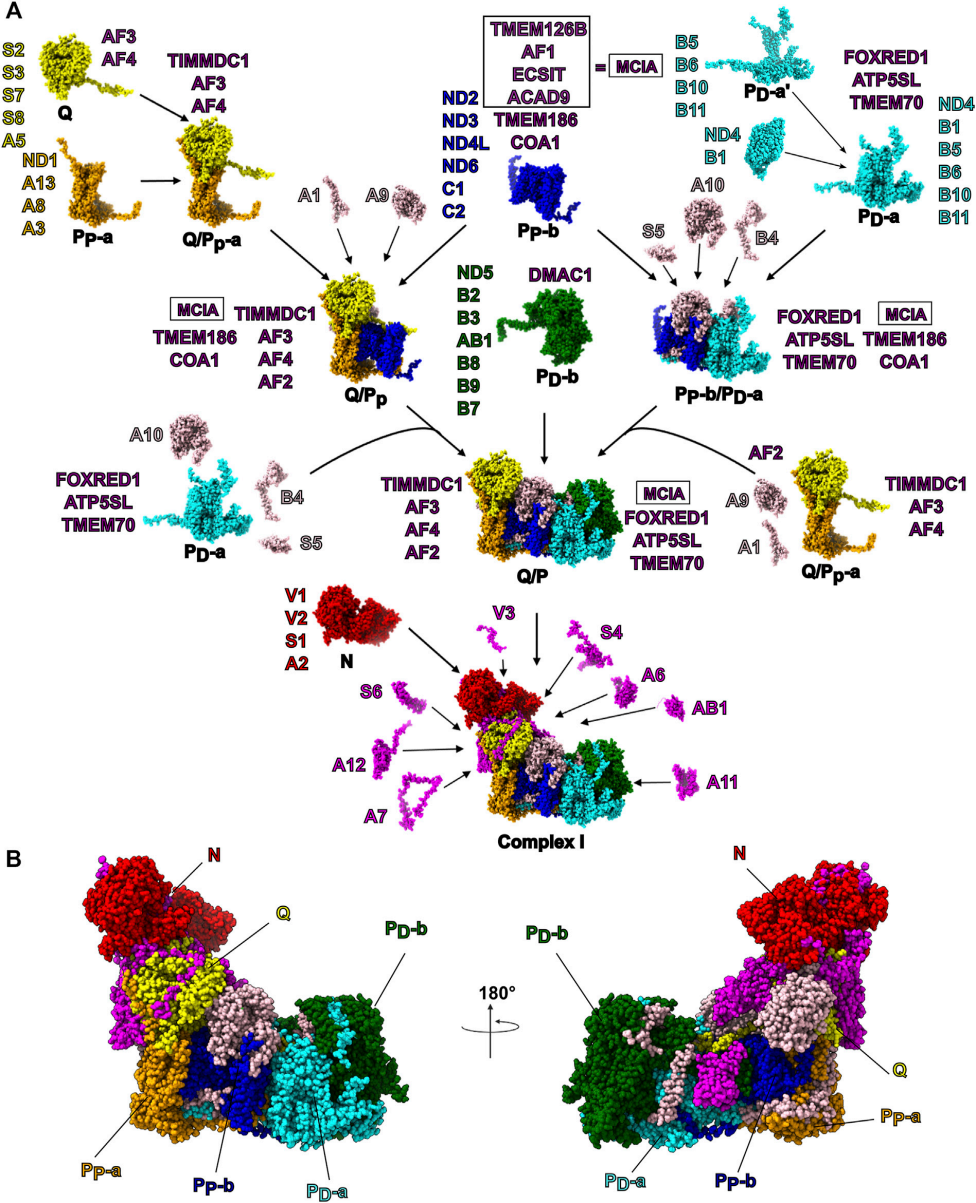
Mammalian CI Assembly. **(A)** The modular assembly pathway of mammalian mitochondrial CI as proposed by Guerrero-Castillo et al. (2017a). The structures of CI subunits within each subassembly are shown as atomic spheres and colored by subassembly. The subunits constituting each subassembly are indicated in shorthand notation (e.g., NDUFS1 is shortened to S1). The assembly factors found associated with each subassembly are indicated in purple. Subunits that associate with intermediate assemblies (e.g., the Q/P_P_ subassembly) are shown in beige. The terminally associated subunits are shown in magenta. **(B)** CI structure shown as atomic spheres with subunits colored by their subassembly as in panel **(A)**. Subunits that associate with intermediate assemblies independent of the modules are shown in beige and terminally associated subunits are shown in magenta.

Mitochondria are the nexus of energy metabolism in eukaryotic cells and play important roles in cellular signaling and apoptosis (Pagliarini and Rutter, 2013). The inner mitochondrial membrane (IMM) harbors the respiratory electron transport chain (ETC) which carries out the final stages of cellular respiration. The ETC is composed of four multi-subunit protein complexes (complex I to complex IV) that couple electron transfers to the pumping of protons across the IMM. The electrochemical proton gradient thus generated is used by ATP synthase (complex V) to generate ATP. Mutations of genes encoding subunits of ETC complexes that result in decreased activity (i.e., ETC deficiencies) are unable to meet the energy demands of muscle and neurons, resulting in severe and often fatal pediatric myopathies and neuropathies (Koene et al., 2012). Dysfunction in complex I (CI) accounts for one third of ETC deficiencies like Leber’s hereditary optic neuropathy, Leigh syndrome and mitochondrial encephalomyopathy (Fiedorczuk and Sazanov, 2018; Ma et al., 2018). The outsized role of CI in mitochondrial disease stems in part from its large number of required subunits and complex assembly pathway (Guerrero-Castillo et al., 2017a). Whereas prokaryotic CI is composed of 14 subunits—7 in the cytoplasm and 7 in the plasma membrane (Brindefalk et al., 2011; Martijn et al., 2018)—mammalian mitochondrial CI is composed of 45 subunits – the 14 “core” subunits conserved from bacteria (Figure 1A) and an additional 31 accessory subunits that have been added during the evolution of eukaryotes (Figure 1B) (Letts and Sazanov, 2015). CI has a boot-shaped structure consisting of a proton-pumping membrane arm that is embedded in the IMM and an electron-transferring peripheral arm extending into the mitochondrial matrix (Figure 1A) (Baradaran et al., 2013; Berrisford et al., 2016). The seven core subunits of the CI membrane arm are encoded by the mitochondrial genome and are conserved across species. The core subunits of the peripheral arm as well as the accessory subunits are encoded by the nuclear genome, expressed in the cytoplasm and imported into the mitochondria. The CI assembly process occurs *via* the successive association of several distinct subassemblies (Figure 2) (Sánchez-Caballero et al., 2016) *via* the action of many essential assembly factors (Guerrero-Castillo et al., 2017a; Formosa et al., 2018). This modular assembly process may provide the organism with additional inputs for regulation and quality control.

Although the core subunits harbor all the substrate binding sites and cofactors needed for catalysis (Baradaran et al., 2013; Berrisford et al., 2016), mutagenesis, knockdown and knockout experiments demonstrate that in eukaryotes the core subunits alone are insufficient for CI assembly and function (Stroud et al., 2013, 2016; Garcia et al., 2017). Given that bacterial CI functions in the absence of accessory subunits, the appearance of essential accessory subunits in eukaryotes suggests changes in the core subunits that make them incapable of operating on their own. However, the roles of each accessory subunit in CI stability, regulation, and function remain unclear. Nonetheless, given that many disease-causing mutations are in the accessory subunits (Fiedorczuk and Sazanov, 2018), it is essential to understand the roles of the accessory subunits to develop treatment strategies for CI deficiencies. Recent advances in membrane-protein biochemistry and cryogenic electron microscopy have led to several high-resolution structures of bacterial and eukaryotic CI (Baradaran et al., 2013; Parey et al., 2019; Bridges et al., 2020; Galemou Yoga et al., 2020; Grba and Hirst, 2020; Kampjut and Sazanov, 2020; Chung et al., 2021; Kolata and Efremov, 2021). CI structures have been observed predominantly in two states: a closed state and an open state which differ in the angle between the peripheral arm and the membrane arm (Fiedorczuk et al., 2016; Zhu et al., 2016; Letts et al., 2019). The open state is characterized by the unfolding of several loops, within the quinone binding cavity and corresponds to the catalytically inert deactive (D) state of CI (Maklashina et al., 2003; Blaza et al., 2018). The closed state corresponds to the active (A) state of the complex (Agip et al., 2018; Letts et al., 2019). In addition, these structures defined the locations of the accessory subunits and advanced our understanding of the mechanism by which CI couples electron transfer to proton pumping (Kampjut and Sazanov, 2020; Parey et al., 2021). However, how the different accessory subunits contribute to CIs assembly and activity, including the transition between the A and D states, remains poorly understood.

In this review, we draw from the recent CI structures, as well as from knockout (Stroud et al., 2013; Stroud et al., 2016; Garcia et al., 2017), assembly (Ugalde et al., 2004; Sánchez-Caballero et al., 2016; Guerrero-Castillo et al., 2017a) and clinical studies (Mimaki et al., 2012; Rodenburg, 2016; Fiedorczuk and Sazanov, 2018) to determine the roles of mammalian CI’s accessory subunits. By comparing structures of the bacteria and eukaryotic core subunits we identify changes that may make the eukaryotic core subunits no longer able to function in the absence of the accessory subunits. Furthermore, by comparing conserved and divergent features of the accessory subunits in mammals, fungi and plants we ascertain the essential structural and functional features of the accessory subunits. We also discuss the structural consequences of disease-causing mutations occurring in the accessory subunits and how they may affect CI function. Overall, we provide a structural and evolutionary framework for understanding the roles of the accessory subunits and propose several experimentally testable hypotheses on their function that we hope will help propel the field towards a full understanding of CI deficiencies and new potential treatments.

Throughout the text we use the human gene names (e.g., NDUFS1, NDUFV1) of the CI subunits, rather than the biochemical names (e.g., 75 kDa subunit, 51 kDa subunit). We also use the full numbering of residues starting from the initiator methionine of the canonical isoform, rather than from the first residue of the mature processed protein. Although the biochemical names have a long and important history in the study of CI, we chose the nomenclature and numbering described above to allow the medical community studying CI deficiencies to make full use of the plethora of structural data without the need for conversion tables. For this review, the accessory subunits were grouped based on their functional implications and discussed roughly starting from the tip of the peripheral arm to the heel of the complex, then from the heel to the tip of the membrane arm along the NDUFA10 side of the complex and finally down the ND5 lateral helix (ND5-HL) side of the membrane arm.

### The Ancient Accessory Subunits of the Peripheral Arm—NDUFS4, NDUFS6 and NDUFA12

**FIGURE 3:**
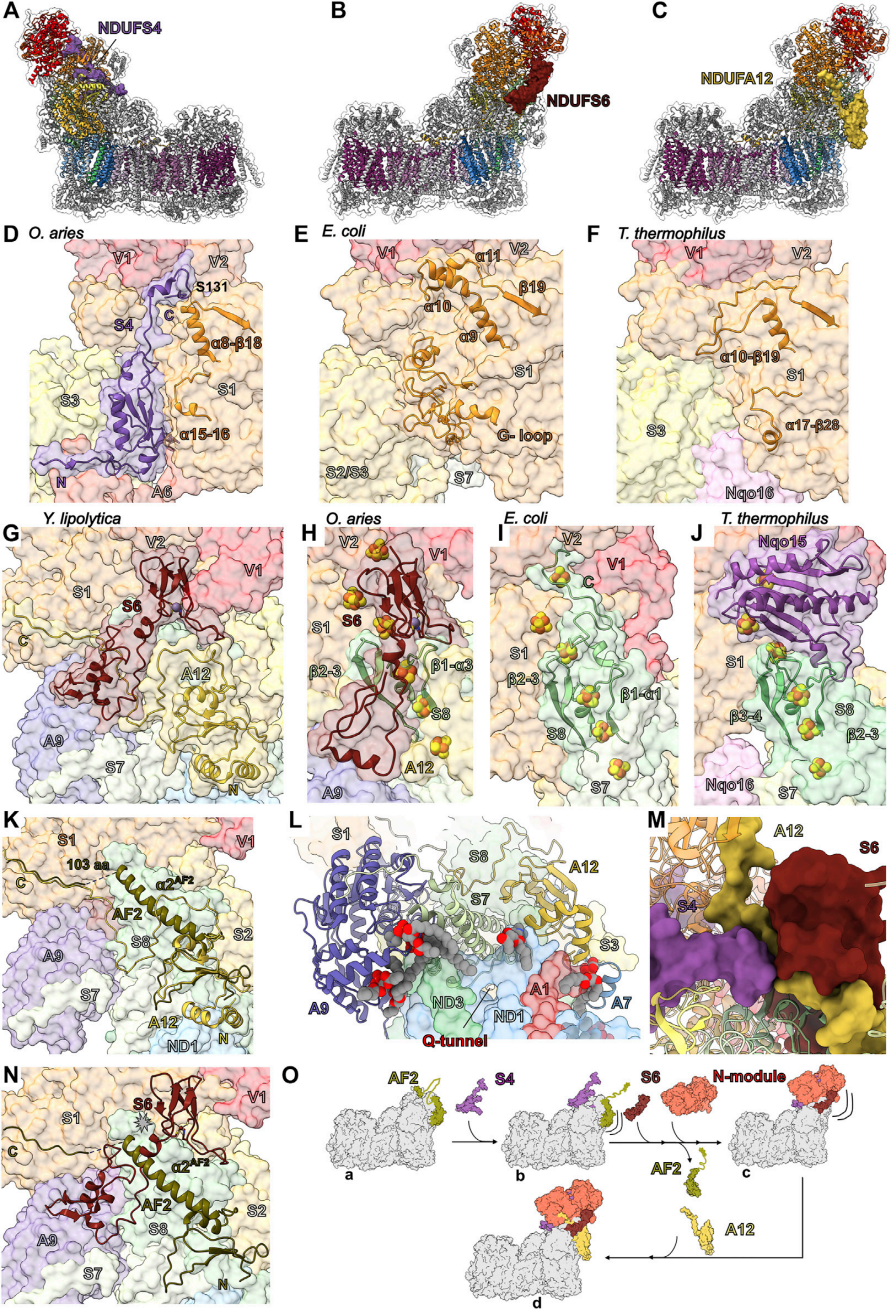
Ancient accessory subunits of CI. **(A)** NDUFS4 (berry), **(B)** NDUFS6 (plum) and **(C)** NDUFA12 (gold) are shown as colored surfaces on CI, the core subunits are shown as colored cartoons and other accessory subunits are shown as grey cartoons. Shin view of **(D)** mammalian (*O. aries* PDB: 6ZKC throughout), **(E)** *E. coli* (PDB: 7NZ1 throughout) and **(F)** *T. thermophilus* (PDB: 4HEA throughout) CI structures shown as transparent surfaces. NDUFS4 and the NDUFS1 loops (orange) that differ between the structures are shown as cartoons. **(G)** Calf view of *Y. lipolytica* CI structure (PDB ID: 6YJ4) shown as transparent surfaces with NDUFS6 (NUMM) and NDUFA12 (N7BM) shown as cartoons with the Zn^2+^ ion shown as atomic sphere and coordinating residues shown as sticks colored by element. Calf view of **(H)** mammalian, **(I)** *E. coli* and **(J)** *T. thermophilus* CI structures shown as transparent surfaces with NDUFS6 and the NDUFS8 (sea green) loops that differ between the structures labeled and shown as cartoons. The FeS clusters and Zn^2+^ ions are shown as atomic spheres and colored by element. **(K)** Structural differences between assembly factor NDUFAF2 and accessory subunit NDUFA12. Calf view of CI showing assembly factor NDUFAF2 from *Y. lipolytica* (PDB: 6RFQ) superposed on NDUFA12 (N7BM) (PDB: 6RFR). The transparent surface of CI is shown with NDUFA12 as cartoon in gold and NDUFAF2 shown as cartoon colored in dark olive. **(L)** Lipid binding around the Q-tunnel. Looking up from the *Y. lipolytica* (PDB: 6YJ4) CI heel showing the accessory subunits NDUFA9 (NUEM) (lavender), NDUFA12 (N7BM) and core subunit NDUFS7 (NUKM) (tea green) as cartoon. Other subunits are shown as transparent surfaces. Lipids are shown as spheres colored by element. **(M)** The interface between NDUFS4 (NUYM), NDUFA12 (N7BM) and NDUFS6 (NUMM) of *Y. lipolytica* CI (PDB: 6RFR). NDUFS4, NDUFA12 and NDUFS6 are shown as surfaces and all other CI subunits are shown as cartoon. **(N)** Clash between NDUFS6 and NDUFAF2. *Y. lipolytica* NDUFAF2 (PDB: 6RFQ) superposed on to NDUFA12 (N7BM) (PDB: 6RFR). NDUFS6 (NUMM) and NDUFAF2 are shown as cartoon. NDUFA12 (N7BM) is not shown for clarity. **(O)** Schematic diagram showing the role of ancient accessory subunits in the “checkpoint” hypothesis of the CI assembly pathway. NDUFS4 binds to CI Q/P subassembly bound to NDUFAF2 and disengages the C-terminal unstructured region of NDUFAF2. Addition of NDUFS6 and N-module to the subassembly results in the release of NDUFAF2 and frees up the NDUFA12 binding site. NDUFA12 binds to the subassembly at the site of NDUFAF2 and completes the assembly. Subunits are colored as in Figure 1 throughout unless stated otherwise. NDUFS4: berry, NDUFS6: plum, NDUFA12: gold, NDUFS1: orange, NDUFS8: sea green, NDUFAF2: dark olive, NDUFA9: lavender, NDUFS7: tea green.

NDUFS6, NDUFA12 and NDUFS4 are ancient accessory subunits present in α-proteobacterial CI (Yip et al., 2011). The α-proteobacteria is the most closely related extant class of bacteria to the mitochondrial ancestor (Martijn et al., 2018), hence, these accessory subunits predate eukaryogenesis (Kahlhöfer et al., 2017) and are conserved throughout eukaryotes. NDUFS4, NDUFS6 and NDUFA12 are assembled at the N/Q interface (Figures 3A–C) during the final stages of CI assembly in mammals (Figure 2A) (Guerrero-Castillo et al., 2017a). Each of these three subunits are important for the activity of CI with many associated disease-causing mutations [recently reviewed by (Kahlhöfer et al., 2021)].

**FIGURE 4:**
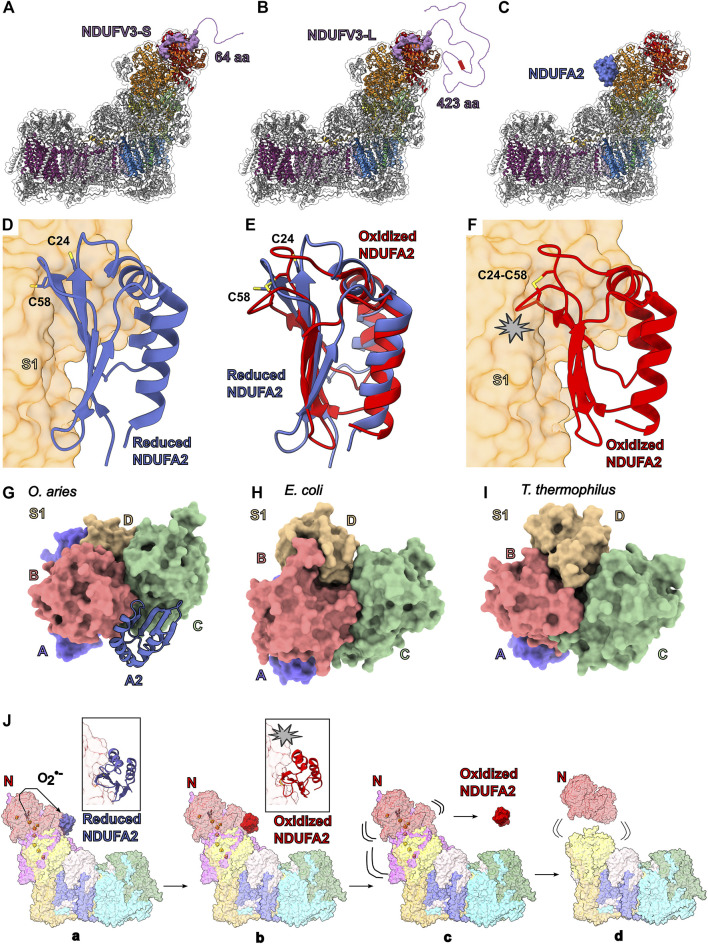
Accessory subunits NDUFV3 and NDUFA2. NDUFV3-S (short isoform) (purple) **(A)**, NDUFV3-L (long isoform) (purple) **(B)** and NDUFA2 (blue) **(C)** are shown as colored surface on CI (PDB: 6ZKC). Disordered N-terminal of the **(A)** short isoform (64 aa) and **(B)** long isoforms (423 aa) are represented by the purple line. Red square in **(B)** represents the conserved string of serine residues. The core subunits are shown as cartoons colored as in Figure 1A and the other accessory subunits are shown as grey cartoon. **(D)** The mammalian reduced NDUFA2 structure shown as cartoon. The cysteines are shown in stick representation and colored by element (PDB: 6ZKC). **(E)** NMR solution structure of oxidized human NDUFA2 (PDB: 1S3A) superposed onto reduced NDUFA2 found in mammalian CI (PDB: 6ZKC). Reduced NDUFA2 is shown in blue cartoon and the oxidized NDUFA2 is shown in red cartoon. **(F)** NMR solution structure of human NDUFA2 (PDB: 1S3A) superposed onto reduced NDUFA2 seen in mammalian CI. The clash caused due to rearrangement of loops [β1-α1^A2^ (aa 23–29) and β2-3^A2^ (aa 57–63)] is shown by the star. **(G-I)** NDUFA2 compensates for the loss of NDUFS1 domain D. Domain architecture of NDUFS1 **(G)** mammalian (*O. aries*) (PDB:5LNK), **(H)** *E. coli* (PDB: 7NZ1) **(I)** *T. thermophilus* (PDB:4HEA) is shown as surfaces colored and labeled according to the domains. **(J)** Schematic representation of the partial dissociation of CI due to oxidation of cysteines in NDUFA2. ROS generated at the FMN site oxidizes the cysteines in NDUFA2 resulting in the formation of a disulfide bond. The rearranged NDUFA2 loop clashes with NDUFS1 resulting in the dissociation of NDUFA2. The dissociation of NDUFA2 destabilizes NDUFS1 and the N-module. This destabilization eventually results in the N-module dissociating. Subunits are colored as in Figure 1 in all the structure figures unless stated otherwise. NDUFV3: purple, reduced NDUFA2: blue, oxidized NDUFA2: red.

*NDUFS4*—In fully assembled CI NDUFS4 is located on the “shin” of the CI boot (Figure 1 and Figure 3A). In eukaryotic CI NDUFS4’s C-terminus binds in a groove between domains A and B of NDUFS1 (Figure 3D and Figure 4G). It is composed of a N-terminal coil, a folded N-terminal domain made up of three *β*-strands, four short α-helices, an extended loop (between β2^S4^ and α3^S4^) and a C-terminal coil that reaches to the N-module and contains a short helix (Figure 3D). NDUFS4’s N-terminal coil interacts with NDUFS3 and NDUFA6 (Figure 3D). The folded N-terminal domain bridges between NDUFS1 in the N-module and NDUFS3 in the Q-module (Figure 3D). The extended β2-α3^S4^ loop reaches across the peripheral arm of the complex interacting with NDUFS1, NDUFS3, NDUFS8, NDUFS6, NDUFA9, NDUFA6 and NDUFA12. NDUFS4’s C-terminal coil interacts with NDUFV1, NDUFV2, NDUFS1 and NDUFV3 (Figure 3D). In mammalian and human cell cultures cAMP promotes the phosphorylation of the NDUFS4 protein at S131^S4^ (Papa, 2002), which is located on NDUFS4’s C-terminal tail. S131^S4^ is buried in a pocket between NDUFS1, NDUFV1 and NDUFV2 that is insufficient to accommodate a phosphate group (Figure 3D). Hence phosphorylation of S131^S4^, which has been proposed to enhance the functional capacity of CI (Papa, 2002), would require a conformational change in this region.

The recent *E. coli* CI structure shows that the groove between A and B domains of NDUFS1 (NuoG) is filled by a large extension from NDUFS1 containing two short helices (α10/α11) (Figure 3E) (Kolata and Efremov, 2021). *E. coli* NDUFS1 also contains a large loop, coined the G-loop, which occupies the equivalent location of NDUFS4’s N-terminal domain in eukaryotes (Figure 3E) (Kolata and Efremov, 2021). In *T. thermophilus*, like in *E. coli*, the NDUFS1 (Nqo3) groove is partly filled with an extended loop (α10-β19 loop) and the accessory subunit Nqo16, not present in *E. coli* or eukaryotes, wedges between NDUFS1, NDUFS3 and NDUFS7 providing additional structural support to this region (Figure 3F) (Baradaran et al., 2013; Berrisford et al., 2016; Kampjut and Sazanov, 2020). As pointed out by Kolata and Efremov (Kolata and Efremov, 2021), these different structural features suggest there is an evolutionary pressure for the stabilization of NDUFS1 by “filling in” this groove. The need for this stabilization has been addressed with three different solutions in the three different CI lineages for which we have structures: the NDUFS4 subunit in the α-proteobacteria/eukaryotes (Figure 3D); the α10/α11 extension and G-loop in the γ-proteobacteria (e.g., *E. coli*) (Figure 3E); and the α10-β19 loop and Nqo16 in the deinococci (e.g., *T. thermophilus*) (Figure 3F). This suggests that in eukaryotes NDUFS4 is needed for the structural integrity of the peripheral arm (Kolata and Efremov, 2021). Consistent with this hypothesis, deletion of NDUFS4 (NUYM) in *Y. lipolytica* (ΔNDUFS4) results in conformational changes in NDUFV1, NDUFV2 and NDUFS1, decreased CI activity and an increase in ROS formation (Kahlhöfer et al., 2017; Parey et al., 2019). Additionally, NDUFS4 is required to protect NDUFS1’s N1b iron-sulfur cluster and ΔNDUFS4 in *Y. lipolytica* exposes this cluster resulting in an altered electron paramagnetic resonance (EPR) signal, likely contributing to the reduction in CI activity (Parey et al., 2019). In the bacterial structures this cluster is protected by the extensions and loops.

*NDUFS6*—In fully assembled CI NDUFS6 is located on the “calf” of the CI boot (Figure 1 and Figure 3B). It is composed of an N-terminal domain with minimal secondary structure and whose fold varies between fungi and mammals (Figures 3G,H) (Parey et al., 2019; Grba and Hirst, 2020; Kampjut and Sazanov, 2020), followed by a coil and short α-helix (α1^S6^) that connects to a C-terminal domain formed by five β-strands forming two small β-sheets (Figure 3H). NDUFS6’s N-terminal domain interacts with NDUFA9, NDUFS4, NDUFA12 and NDUFS8 (Figure 3H). The interdomain coil and the short helix of NDUFS6 lodge in a cleft on the surface of NDUFS8 formed by the β1-α3^S8^ and β2-3^S8^ loops (Figure 3H). The C-terminal domain binds a Zn^2+^ ion and sits in a pocket formed by core subunits NDUFS1, NDUFV1, NDUFV2 and NDUFS8 (Figure 3H). In the structures of plant and algal CI (Maldonado et al., 2020; Soufari et al., 2020; Klusch et al., 2021), NDUFS6 lacks the N-terminal domain altogether and the sequence for the N-terminal domain is missing from the α-proteobacterial CI, indicating that this domain evolved later in specific eukaryotic lineages.

On the “calf” side of CI where NDUFS6 is located in mammals, the *T. thermophilus* and *E. coli* CIs have extended loops/coils—the NDUFS8 (Nqo9) β3-4^S8^ loop in *T. thermophilus* and the extended C-terminal coils of NDUFS8 (NuoI), NDUFS7 (NuoB) and NDUFV1 (NuoF) in *E. coli* – bind overtop of the NDUFS1 (NuoG) FeS clusters (Figures 3I,J) (Baradaran et al., 2013; Berrisford et al., 2016; Kolata and Efremov, 2021). In mammalian CI, the corresponding loops are shorter and the extended loops in the bacterial structures would conflict with the location of NDUFS6 (Figure 3H). This suggests that NDUFS6 replaces these bacterial sequences and acts to stabilize the NDUFS1 FeS clusters. Indeed, NDUFS6 (NUMM) deletion (ΔNDUFS6) or mutation of a NDUFS6 Zn^2+^ coordinating cysteine in *Y. lipolytica*, showed loss of EPR signal for nearby cluster N4, indicating severe disruption (Kmita et al., 2015). In humans, the mutation of one of the Zn^2+^ coordinating cysteines (C115Y^S6^) results in fatal neo-natal lactic acidosis (Kmita et al., 2015) indicating the importance of NDUFS6’s Zn^2+^ coordination in stabilizing its interaction with CI. It has been noted (Kmita et al., 2015) that two other Zn^2+^ containing proteins, IscU (Markley et al., 2013) and mito-NEET (Zuris et al., 2011), with the same Zn^2+^ coordination pattern as NDUFS6 (three-cysteine/one-histidine), are involved in FeS biogenesis. This suggests that NDUFS6 may have initially started as an assembly factor for the FeS clusters of the peripheral arm that became permanently associated with the complex as a stabilizing subunit.

*NDUFA12*—Located on the “calf” of the CI boot below NDUFS6, NDUFA12 sits at the interface of the matrix and IMM (Figure 1 and Figures 3C,G). It is composed of a structured N-terminal domain that binds to the Q-module adjacent to the membrane and a C-terminal coil which extends to the N-module forming an extensive interface with the NDUFS8 and crossing under the N-terminal domain of NDUFS6 (Figure 3G). This suggests that either NDUFA12 binds the complex first, that NDUFS6 and NDUFA12 pre-assemble and bind together or that conformational changes in NDUFS6 are needed for NDUFA12 binding (Parey et al., 2019). The N-terminal domain consists of two amphipathic α-helices that interact with lipid, followed by a three stranded *β*-sheet, and an α-helix and interacts with NDUFS8, NDUFA7, NDUFS2 and NDUFS7 (Figures 3G,K). The amphipathic helices play a role, along with NDUFA9 and NDUFS7, in distorting the membrane in the region of the CoQ-tunnel (Figure 3L) (Parey et al., 2019).

In *Y. lipolytica*, deletion of NDUFS6 (ΔNDUFS6) results in the assembly factor NDUFAF2 remaining bound to the enzyme and a decrease in the bound quinone content of the complex relative to wildtype (Parey et al., 2019). NDUFA12 and NDUFAF2 are paralogs and occupy the same binding site on CI (Parey et al., 2019), thus, NDUFAF2 must be removed from the assembly intermediate before NDUFA12 can bind (Vogel et al., 2007). Importantly, NDUFAF2 lacks the amphipathic helices seen in NDUFA12 (Figure 3K) and therefore fewer bound lipids are seen at the interface in the ΔNDUFS6 structure (Parey et al., 2019). The observation of decreased CoQ-content is consistent with the hypothesis that deformation of the membrane by NDUFA12, NDUFA9 and NDUFS7 promotes CoQ access to the CoQ-tunnel (Figure 3L) (Parey et al., 2019). Reduced CI activity seen in the NDUFA12^KO^ (Stroud et al., 2016) and a naturally occurring NDUFA12 nonsense mutation (Ostergaard et al., 2011) may be due in part to less efficient CoQ access to the active site. The inability of NDUFAF2 to deform the membrane may help prevent reverse electron transport (RET), which can generate significant reactive oxygen species (ROS) (Chouchani et al., 2014), before complete assembly of the complex at which point NDUFAF2 is replaced by NDUFA12 and CI is activated.

*Roles of NDUFS4, NDUFS6 and NDUFA12 in CI assembly—*Together NDUFS4, NDUFS6 and NDUFA12 play an important role in the attachment of the N-module during CI assembly. Patients with deletion or substitution mutations in the *NDUFS4* gene show an abnormal assembly profile with a complete loss of the fully assembled CI (Assouline et al., 2012). NDUFS4^KO^ studies in mice suggest that CI is unstable or fails to assemble properly in the absence of NDUFS4 (Kruse et al., 2008). In HEK293T cells, although, NDUFS4 only interacts with the C-terminal coil of NDUFA12 over a short sequence (Figure 3M), NDUFS4^KO^ results in poor incorporation of NDUFA12 but overall only mild assembly defects (Calvaruso et al., 2012; Stroud et al., 2016). The dependence on NDUFS4 for the incorporation of NDUFA12 was not seen in *Y. lipolytica* ΔNDUFS4 as this structure showed clear incorporation of NDUFA12 (Parey et al., 2019). However, although assembly goes to completion and NDUFA12 is incorporated into the complex, ΔNDUFS4 resulted in an increase of bound assembly factor NDUFAF2 (Parey et al., 2019; Kahlhöfer et al., 2021) indicating that the deletion introduces a bottleneck in the final stages of assembly (Kahlhöfer et al., 2021).

In HEK293T cells, NDUFS6^KO^ prevents attachment of the N-module, stalling CI assembly at the Q/P subassembly (Figure 2A) (Stroud et al., 2016; Guo et al., 2017). In *Y. lipolytica*, ΔNDUFS6 leads to accumulation of a CI intermediate with the assembly factor NDUFAF2 bound, lacking both NDUFS6 and NDUFA12 but with the N-module attached (Kmita et al., 2015; Parey et al., 2019). NDUFAF2 lacks the N-terminal amphipathic α-helices seen in NDUFA12, but has a similar central domain followed by a NDUFAF2 specific α-helix (α2^AF2^) (Figure 3K) (Parey et al., 2019). The C-terminal region of NDUFAF2 contains a disordered region of 103 amino acid residues followed by a coil that binds in a similar position as the C-terminal coil of NDUFA12 (Figure 3K). The α2^AF2^ helix binds to the surface of NDUFS8 where NDUFS6 would normally bind, indicating that when bound to CI, NDUFAF2 would prevent NDUFS6 binding (Figure 3N) (Kmita et al., 2015; Parey et al., 2019). Given that the *NDUFA12* gene is wild type in the ΔNDUFS6 *Y. lipolytica* strain, but NDUFA12 is not seen on CI, NDUFA12 alone is not sufficient to remove NDUFAF2 and NDUFS6 must play an important role in the removal of NDUFAF2 during the final stages of CI assembly. The difference in N-module attachment between NDUFS6^KO^ in HEK293T cells and ΔNDUFS6 in *Y. lipolytica* indicate a more severe impact of loss of NDUFS6 on CI assembly in mammals compared to yeast.

In HEK293T cells, NDUFA12^KO^ increases the abundance of CI subassemblies and decreases CI activity, however, fully assembled CI is still observed (Stroud et al., 2016). In humans, a NDUFA12 nonsense mutation leads to a similar phenotype, i.e., although CI appears fully assembled in patients with the mutation, they have reduced CI activity resulting in Leigh syndrome (Ostergaard et al., 2011). These data indicate that, in contrast to the loss of NDUFS6, the loss of NDUFA12 does not prevent the full assembly of the complex but negatively impacts CI turnover.

From the wild type and mutant CI studies, the hypothesis arises that NDUFAF2 acts in concert with NDUFS4, NDUFS6 and NDUFA12 as an assembly checkpoint, blocking the full assembly of the peripheral arm until the rest of the complex is fully assembled. In this scenario, the formation of the Q/P subassembly (Figure 2A), which contains the full membrane arm and Q-module along with NDUFAF2, promotes the association of NDUFS4, which in turn recruits the N-module and NDUFS6. As pointed out by Parey et al., (2019) NDUFS4 binding also likely promotes exchange of NDUFAF2 for NDUFA12 *via* its extended β2-3^S4^ loop (Figure 3M).

This “checkpoint” hypothesis differs from the model proposed by Parey et al., (2019), in which NDUFAF2 recruits the N-module to the Q/P subassembly, in two major ways. 1) Given the inability of smaller NDUFAF2 containing assembly intermediates to recruit the N-module, NDUFAF2 alone is insufficient for N-module recruitment. Thus, NDUFS4 and potentially NDUFS6 are required to help bind the N-module (Figure 3O). Importantly, in mammals NDUFS4 and NDUFS6 do not associate with the complex until after formation of the Q/P subassembly preventing premature attachment of the N-module (Figure 2A). 2) Given that NDUFS6 is required for the removal of NDUFAF2, interaction of NDUFS6 with the complex likely precedes that of NDUFA12, whose binding site is only available after NDUFAF2 is removed (Figure 3O). This necessitates a more complicated “dance” in which the C-terminus of NDUFA12 works its way under the N-terminal domain of NDUFS6 and likely involves additional intermediate conformations (Figure 3O).

Notably, in plant mitochondrial CI, NDUFS6 lacks the N-terminal domain (Maldonado et al., 2020; Soufari et al., 2020; Klusch et al., 2021). This would simplify the assembly process for the peripheral arm, as the NDUFA12 C-terminal coil no longer passes under NDUFS6 (Maldonado et al., 2020). Consistent with the interactions of these subunits regulating the association of the N-module, plant CI assembles by a distinct pathway, in which the N-module is connected to the complex before the P_D_-module, forming the plant specific assembly intermediate CI* (Ligas et al., 2019; Maldonado et al., 2020). Although possible genes for NDUFAF2 have been identified in plants, biochemical studies of CI assembly in plant have yet to identify NDUFAF2 as a plant CI assembly factor (Meyer et al., 2019). Nonetheless, if overcoming the checkpoint for N-module attachment is initiated by NDUFS4 binding, the major difference between plants and opisthokonts could be the dependence of NDUFS4 binding on the attachment of the P_D_-module.

The variety in assembly pathways suggests that the specific roles of NDUFS4, NDUFS6 and NDUFA12 in regulating CI assembly may have evolved later and that the original physiological roles of these subunits are structural. In α-proteobacteria the NDUFS6 gene lacks the sequence for an N-terminal domain and assembly of CI in *E. coli* follows a more “plant-like” pathway (though *E. coli* lacks the accessory subunits) (Friedrich et al., 2016). Further studies on CI assembly and structure in the α-proteobacteria would shed more light on these issues (Jarman et al., 2021).

### The Subunit at the Tip of the Peripheral Arm: NDUFV3

NDUFV3 lies on the tip of the N-module and joins the complex in the final stage of assembly (Figure 2A and Figure 4A). NDUFV3 is composed of a N-terminal coil, an α-helix and a C-terminal coil that bind in the cleft between NDUFV1 and NDUFV2. The C-terminal coil of NDUFV3 reaches to NDUFS1 where it interacts with the C-terminal coil of NDUFS4. NDUFV3 is known to be present on CI in two isoforms (Figures 4A,B) (Bridges et al., 2017; Dibley et al., 2017; Guerrero-Castillo et al., 2017b). The 10 kDa “short” isoform (NDUFV3-S) is found in heart tissue while the 50 kDa “long” isoform (NDUFV3-L), generated by alternative splicing of the *NDUFV3* gene, appears to be dominant in other tissues (liver and brain) and in HEK293T cells (Bridges et al., 2017; Dibley et al., 2017). NDUFV3^KO^ in HEK293T cells showed that lack of both NDUFV3 isoforms led to only minor defects in the assembly and function of CI suggesting its presence in CI is not essential (Stroud et al., 2016; Dibley et al., 2017). In addition, it was found that NDUFV3-S alone is sufficient for CI assembly (Dibley et al., 2017). The N-terminal 64 amino acid residues of NDUFV3-S are disordered and the region of NDUFV3-S that is bound to NDUFV1 and NDUFV2 and clearly resolved in the structure is conserved in NDUFV3-L, suggesting they bind similarly to CI (Figures 4A,B). Therefore, the main difference between the isoforms is not in their interaction with CI but that NDUFV3-L has an additional 423 amino acid residues on its N-terminus that are also predicted to be disordered (Figures 4A,B) (Guerrero-Castillo et al., 2017b; Bridges et al., 2017; Dibley et al., 2017).

Phylogenic tree analysis has shown that NDUFV3 is absent in jawless fish, insects, nematodes, and fungi suggesting it has evolved within the vertebrates (Bridges et al., 2017). Further, a sequence alignments show conservation in the C-terminal structured region of both NDUFV3 isoforms as well as in a string of 10 serine residues only present in NDUFV3-L (Figure 4B). Phospho-proteome analyses have identified three serine residues, from the string of 10, to be phosphorylated in various conditions which is consistent with the previous identification of NDUFV3-L as a mitochondrial phosphoprotein (Bridges et al., 2017).

Interestingly, as noted by Bridges et al. (2017), the size, lack of secondary structure and binding site of NDUFV3-L resembles the 68 kDa fragment of the atypical cadherin (Ft4) which has been shown to regulate CI activity in *Drosophila melanogaster* (Sing et al., 2014). Fat (Ft) cadherins are cell adhesion molecules that control tissue growth and organization (Tanoue and Takeichi, 2005). In *Drosophila*, Ft regulates ETC integrity and promotes OXPHOS by release of a soluble 68 kDa fragment by proteolytic cleavage of its intracellular domain, which is imported into mitochondria and binds to CI at NDUFV2 (Sing et al., 2014). Loss of Ft in *Drosophila* leads to loss of CI and increases ROS (Sing et al., 2014). This led Bridges et al. (2017) to propose that NDUFV3-L may play a similar role in mammals. Given that the unstructured regions of the intracellular domains of cadherins are known to associate with a variety of adaptors and signaling proteins (Maître and Heisenberg, 2013) and that unstructured regions of proteins are commonly involved in protein-protein interactions (Dyson and Wright, 2005), it is reasonable to speculate that NDUFV3-L has additional interaction partners and that the physiological impact of the NDUFV3 isoforms may have more to do with those additional interactions than with possible changes in the behavior of CI itself. In this scenario, CI is used as a scaffold for the localization of NDUFV3 to the matrix surface of the cristae. More work is needed to identify any additional NDUFV3 interaction partners and determine what role it may be playing at the cristae surface.

### The Thioredoxin Fold Subunit NDUFA2

NDUFA2 is assembled into the N-module subassembly along with NDUFV1, NDUFV2 and NDUFS1 prior to attachment to CI (Figure 2A and Figure 4C) (Sánchez-Caballero et al., 2016; Guerrero-Castillo et al., 2017a). NDUFA2 has a thioredoxin fold consisting of a β-sheet with four anti-parallel β-strands and three α-helices (Figure 4D). Despite the structural homology to thioredoxins, the two vicinal cysteines of the canonical thioredoxin CXXC motif are not conserved, instead NDUFA2 has two different cysteines (Cys24^A2^ and Cys58^A2^ in humans) conserved across eukaryotes (with a notable exception of Ala47^A2(NI8M)^ in *Y. lipolytica*).

In all current structures of eukaryotic CI, NDUFA2 interacts solely with core subunit NDUFS1 (Figures 4C,D) in a reduced form i.e., there is no disulfide bond between the conserved cysteine residues (Figure 4D). Nonetheless, the structure of isolated human NDUFA2 in the oxidized state, containing a disulfide linkage between Cys24^A2^ and Cys58^A2^, has been solved by NMR (Figure 4E) (Brockmann et al., 2004). In the oxidized state NDUFA2 adopts a distinct conformation compared to the reduced state (Figure 4E). When docked onto the structure of CI the oxidized conformation of NDUFA2 loops clashes with the surface of NDUFS1 (Figure 4F). This indicates that change in reduction state of NDUFA2 would impact NDUFA2’s interaction with NDUFS1.

In bacteria, NDUFS1 (Nqo3 in *T. thermophilus* and NuoG in *E. coli*) is composed of four large domains A-D (Figures 4H,I). However, in eukaryotes, NDUFS1 lacks a significant domain D, and thus is composed of domains A-C alone and a short C-terminal helix (Figure 4G). In bacteria, domain D binds at the interface of domains B and C and likely stabilizes the overall domain architecture of NDUFS1 (Figures 4H,I). NDUFA2 in eukaryotes binds on the opposite face of NDUFS1 relative to domain D in bacteria, but like domain D in bacteria, NDUFA2 bridges domains B and C (Figure 4G). Thus, it is reasonable to hypothesize that, like domain D of bacteria, NDUFA2 stabilizes the overall domain architecture of NDUFS1, thereby compensating for the lack of domain D. Thus, the stability of eukaryotic NDUFS1 may depend on the presence of NDUFA2, thereby making the eukaryotic CI N-module dependent on this accessory subunit. Consistent with this NDUFA2^KO^ in HEK293T cells results in the accumulation of a CI intermediate lacking the N-module and severe defects in CI activity and respiration (Stroud et al., 2016).

These observations lead to the hypothesis that NDUFA2 may act as a ROS sensor that works to “shut off” CI activity under conditions of high ROS production. In this scenario, molecules of superoxide or hydrogen peroxide produced at the FMN site of CI could react with the conserved cysteines of NDUFA2, stripping electrons from them and resulting in the formation of a disulfide bond (Figure 4J). This would result in the conformational rearrangement of the NDUFA2 loops causing it to clash with NDUFS1 and unbind (Figure 4J). Loss of NDUFA2 would destabilize the NDUFS1 structure by removing the bridging interactions between domains B and C impacting NDUFS1’s interaction with other N- and Q-module subunits (Figure 4J). In short, reaction of NDUFA2 with ROS may be an initiating factor resulting in the observed partial degradation of CI under RET conditions (Guarás et al., 2016). If correct, this hypothesis makes several experimentally testable predictions. First, treatment of isolated CI with oxidizing agents should result in loss of NDUFA2 followed by loss of the N-module. Second, *Y. lipolytica* CI, due to its lack of one of the conserved NDUFA2 cysteines, should be more resistant to oxidizing agents.

### The Lone Accessory Subunit of the Q Subassembly: NDUFA5

**FIGURE 5:**
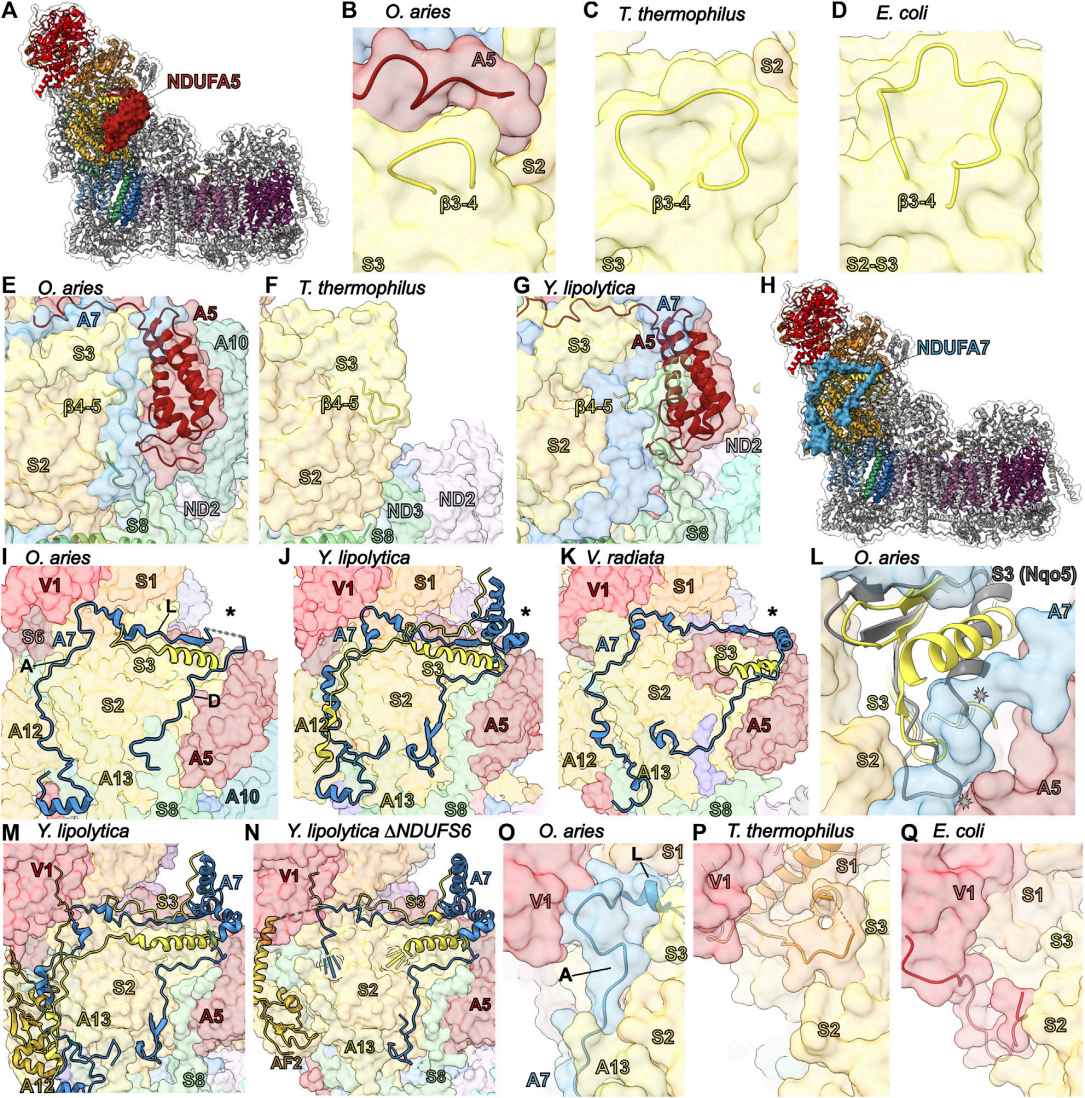
Accessory subunits NDUFA5 and NDUFA7. **(A)** The structure of CI (PDB: 6ZKC) in cartoon with the surface of NDUFA5 (auburn) shown, the core subunits colored as in Figure 1A and the accessory subunits grey. **(B–D)** The NDUFA5 C-terminus compensates for a shorter NDUFS3 (corn yellow) β3-4^S3^ loop. **(B)** Mammalian (PDB: 6ZKC)*,* **(C)** *T. thermophilus* (PDB: 4HEA) and **(D)** *E. coli* (PDB: 7NZ1). CI structures shown in transparent surface with NDUFA5 and the NDUFS3 β3-4^S3^ loop in cartoon. **(E–G)** NDUFA5 may compensate for a shorter NDUFS3 β4-5^S3^ loop. **(E)** *O. aries* cartoon of NDUFS3 β4-5^S3^ loop (aa 99–102), NDUFA5, and NDUFS8 (sea green) on the CI surface (PDB: 6ZKC). The β4-5^S3^ loop is shorter in *O. aries* than in bacteria. **(F)** *T. thermophilus* cartoon of the β4-5^S3^ loop (aa 76–86) and NDUFS8 (Nqo9) on the CI surface (PDB: 4HEA). **(G)** *Y. lipolytica* cartoon of the NDUFS3 β4-5^S3^ (NUGM) loop (aa 147–150), NDUFA5 (NUFM) and NDUFS8 (NUPM) on the CI surface (PDB: 6YJ4). *Y. lipolytica* has an extended NDUFS8 coil compared to *O. aries* and *T. thermophilus*. **(H)** The structure of CI (PDB: 6ZKC) shown in cartoon with the surface of NDUFA7 (azzure blue), the core subunits colored as in Figure 1 and the accessory subunits grey. **(I–K)** Differences in NDUFA7 between eukaryotes. **(I)** *O. aries* NDUFA7 shown as cartoon on the CI (PDB: 6ZKC) surface with the variable region indicated (*) and the N-terminal coil and helix of NDUFS3 shown as cartoon. A: ascending coil, L: lateral coil, D: descending coil. **(J)** *Y. lipolytica* NDUFA7 (NUZM) shown as cartoon on the CI (PDB: 4HEA) surface with the variable region indicated (*) and the N-terminal coil and helix and C-terminal coil of NDUFS3 (NUGM) shown as cartoon. **(K)** *V. radiata* NDUFA7 (NDUA7) shown as cartoon on the CI (PDB: 6X89) surface with the variable region indicated (*) and the N-terminal coil and helix of NDUFS3 (NDUS3) shown as cartoon. **(L)** A conformation change is needed in the N-terminal helix of NDUFS3 relative to that from *T. thermophilus* to accommodate NDUFA7. Cartoon of NDUFS3 in *O. aries* (PDB: 6ZKC) and NDUFS3 (Nqo5; grey) in *T. thermophilus* (PDB: 4HEA) on the *O. aries* CI surface. **(M,N)** NDUFA7 participates in NDUFA12 (gold) binding. **(M)** Cartoon of NDUFA7 (NUZM), the N-terminal and C-terminal loop of NDUFS3 (NUGM) and NDUFA12 (N7BM) on the CI surface (PDB: 6YJ4). **(N)** Cartoon of NDUFA7, NDUFS3 and NDUFAF2 (N7BML) (gold) on the CI surface (PDB: 6RFQ). The N-terminal regions of NDUFA7 and NDUFS3 are disordered. **(O–Q)** Stabilizing role for NDUFA7 at the interface of the N and Q modules. **(O)** *O. aries* cartoon of NDUFA7 on CI surface (PDB:6ZKC). NDUFA7 stabilizing interactions are replaced by core subunits in bacteria. A: ascending coil, L: lateral coil. **(P)** *T. thermophilus* cartoon of NDUFS1 (orange) (Nqo1) on CI surface (PDB: 4HEA). **(Q)** *E. coli* cartoon of C-terminal coil of NDUFV1 (NuoF) on CI surface (PDB: 7NZ1). Subunits are colored as in Figure 1 throughout unless stated otherwise. NDUFA5: auburn, NDUFS3: corn yellow, NDUFS8: sea green, NDUFA7: azzure blue, NDUFA12: gold, NDUFS1: orange.

NDUFA5 binds at the interface of core subunits NDUFS2 and NDUFS3 and is composed of a short N-terminal coil, a three-helix bundle, and a long C-terminal coil (Figures 5A,E). Along with NDUFS2, NDUFS3, NDUFS7 and NDUFS8, NDUFA5 forms the Q subassembly (Figure 2A). In the fully assembled complex, the N-terminal coil and three-helix bundle of NDUFA5 interacts with NDUFS2, NDUFS3 and NDUFA7 (Figure 5E). The three-helix bundle also interacts with NDUFA10 in a state-dependent manner (discussed in the section on NDUFA10). The C-terminal loop of NDUFA5 interacts with NDUFS2, NDUFS3 and NDUFS4. In HEK293T cells, NDUFA5^KO^ results in incomplete assembly of CI with the accumulation of a 460 kDa subcomplex composed of membrane arm subunits but lacking any Q- or N-module subunits (Stroud et al., 2016). In addition, HEK293T cells with NDUFA5 depleted by RNA interference showed a significant decrease in CI activity compared to other complexes (Rak and Rustin, 2014). *Ndufa5* deletion in mice is embryonic lethal and conditional neuronal-specific KO in mice yielded mild chronic encephalopathy with concomitant decreases in the levels of fully assembled CI and CI activity (Peralta et al., 2014). Together, these data indicate that NDUFA5 is required for the stability of the Q-module and the formation of a functional CI.

Bacterial CI structures, both *T. thermophilus* and *E. coli*, contain extended loops in NDUFS3 (Nqo5 and NuoCD, respectively), that would conflict with the position of NDUFA5 in eukaryotes (Figures 5B–F). Importantly, in *E. coli*, but not in *T. thermophilus*, the NDUFS2 and NDUFS3 subunits are a single polypeptide (NuoCD). Nonetheless, in both bacterial structures, an extended β3-4^S3^ loop (β3-4^Nqo5^ and β3-4^NuoCD^) conflicts with the position of the NDUFA5 C-terminal coil in the mitochondrial CI structures (Figures 5B–D). This extended loop provides additional contacts between NDUFS3 and NDUFS2 in *T. thermophilus* and would stabilize the overall NuoCD fold in *E. coli*. Thus, NDUFA5 in mammals would replace these lost bacterial stabilizing interactions between NDUFS2 and NDUFS3. Additionally, the loop corresponding to β4-5^S3^ in *T. thermophilus* (β4-5^Nqo5^) is also longer with an extended interaction interface between NDUFS3 and NDUFS2 (Figure 5F). This extended β4-5^S3^ loop would also conflict with the position of NDUFA5 in mitochondrial CI (Figures 5E,G). In *Y. lipolytica*, an additional stabilizing interaction involving the N-terminal coil of NDUFS8 is seen in this region, bridging NDUFA5 and NDUFS3 (Figure 5G). Overall, the differences of the NDUFS2 and NDUFS3 interfaces between the bacteria and mitochondrial structures indicate that different strategies have evolved to support the association of these subunits. On one extreme there is *E. coli* that uses a fused NuoCD subunit and on the other is eukaryotes that have recruited NDUFA5 to facilitate interaction between NDUFS2 and NDUFS3. In the middle is *T. thermophilus* that has extended loops that help facilitate the interaction and occupy the equivalent position of NDUFA5 in eukaryotes.

Nonetheless, differences in the interaction interface between NDUFS2 and NDUFS3 in mammalian and *T. thermophilus* CIs alone cannot explain the dependency on NDUFA5 for Q-module assembly. In human CI the interface between NDUFS2 and NDUFS3 is larger (2,747.1 Å^2^ vs. 2,613.5 Å^2^) and more energetically favorable (-20.2 kcal/mol vs. -16.6 kcal/mol) than that of *T. thermophilus* (Krissinel and Henrick, 2007)*.* This suggests that NDUFA5 binding, in addition to providing stabilizing interactions between the subunits, may also allosterically regulate their interaction, promoting necessary conformational changes that allow for Q-module assembly. Interaction studies between isolated NDUFS2 and NDUFS3 in the presence and absence of NDUFA5 are needed to address this hypothesis.

### The Square Coil of the Peripheral Arm: NDUFA7

NDUFA7 forms part of the Q-module and it is believed to be among the last subunits added as no intermediates containing this subunit have been observed (Figure 2A) (Sánchez-Caballero et al., 2016). Except for the N-terminal amphipathic helix, which binds at the interface of the matrix and IMM adjacent to NDUFS8, NDUFA1 and NDUFA12, most of NDUFA7 is an extended coil without secondary structure that ascends from the membrane to the N-module, continues laterally along the N/Q-module interface then descends back towards the membrane (Figures 5H,I). NDUFS8, NDUFA12 and NDUFS2 create a binding surface for the NDUFA7 “ascending” coil (Figure 5I). The NDUFA7 “lateral” coil interacts with NDUFV1, NDUFS1 and NDUFS2 (Figure 5I). The lateral coil is followed by a variable region that is disordered in mammals (residues 72–89), a small α-helical domain in *Y. lipolytica* and a short α-helix in plants (Figures 5I–K) (Guo et al., 2017; Grba and Hirst, 2020; Kampjut and Sazanov, 2020; Maldonado et al., 2020). This variable region interacts with NDUFS3 and NDUFA5. The C-terminal NDUFA7 “descending” coil extends towards the N-terminus of NDUFS8 interacting with NDUFS2, NDUFS3 and NDUFA5 (Figure 5I). Adjacent to the variable region the lateral and descending coils sandwich the N-terminal α-helix of NDUFS3 resulting in a significant reorientation of the NDUFS3 helix in eukaryotic CI relative to its position in the bacterial structures (Figure 5L).

In HEK293T cells, NDUFA7^KO^ has negligible to moderate reductions in CI activity and stability. However, in any accessory subunit KO that results in the reduction of N-module incorporation, e.g., NDUFS6^KO^, NDUFA2^KO^ and NDUFA6^KO^, leads to a reduction of NDUFA7 (Stroud et al., 2016). This suggests that although NDUFA7 interacts mostly with Q-module subunits it requires the N-module for binding and has resulted in the proposal that NDUFA7 should be considered a N-module, as opposed to Q-module, subunit (Formosa et al., 2018). In cardiac cells, depletion of NDUFA7 promotes ROS generation and calcineurin signaling activation that results in the expression of cardiac hypertrophy genes (Shi et al., 2020).

Interestingly, in the *Y. lipolytica* ΔNDUFS6 CI structure – in which the assembly factor NDUFAF2 is bound instead of NDUFA12—the NDUFA7 amphipathic helix and ascending coil along with the N-terminal coil of NDUFS3—which binds overtop of the NDUFA7 ascending coil in *Y. lipolytica*—are disordered (Figures 5M,N). This indicates that full binding of NDUFA7 likely requires the exchange of NDUFAF2 for NDUFA12 during attachment of the N-module. However, in *Y. lipolytica* the C-terminal coils of NDUFA7 are held in place by the interaction between the small α-helical domain of the variable region and the C-terminal coil of NDUFS3 that wraps over the lateral coil, as well as additional interactions with the N-terminus of NDUFS8 (Figures 5G,J,M,N). These interactions are missing in mammals—which lack the α-helical domain and have shorter NDUFS3 C-terminal and NDUFS8 N-terminal coils (Figure 5I)—suggesting that NDUFA7 binding may be more sensitive to the exchange of NDUFAF2 and NDUFA12 in mammals.

Outside of the N-terminal amphipathic helix the most conserved region of NDUFA7 is the “corner” between the ascending and lateral coils which binds at the interface of NDUFV1 and NDUFS1 of the N-module and NDUFS2 and NDUFS3 of the Q-module (Figure 5O). In *T. thermophilus* and *E. coli* CIs this interface is filled with the NDUFS1 α3-β6^S1^ loop and the N-terminal coil of NDUFV1, respectively, which are much longer than their eukaryotic counterparts (Figures 5P,Q) (Baradaran et al., 2013; Kolata and Efremov, 2021). Like in the case of NDUFS4, these different interactions represent three different strategies for stabilizing this interface in the three different CI lineages (Figures 5O–Q). In mammals, it has also been shown that NDUFA7 is a target for phosphorylation by cAMP-dependent protein kinase (mtPKA) at Ser95^A7^ located at the interface of NDUFS3 and NDUFA5 (Rak and Rustin, 2014). Analogous to the interaction with the NDUFS3 C-terminal coil in *Y. lipolytica*, phosphorylation may promote NDUFA7 binding in this region and hence could play a part in regulating CI assembly (Palmisano et al., 2007). This leads to the hypothesis that NDUFA7, although not essential, functions to stabilize the attachment of the N-module and may aid in the replacement of NDUFAF2 with NDUFA12 during assembly.

### The LYR/Acyl Carrier Protein Pairs: NDUFA6/NDUFAB1-α and NDUFB9/NDUFAB1-β

**FIGURE 6:**
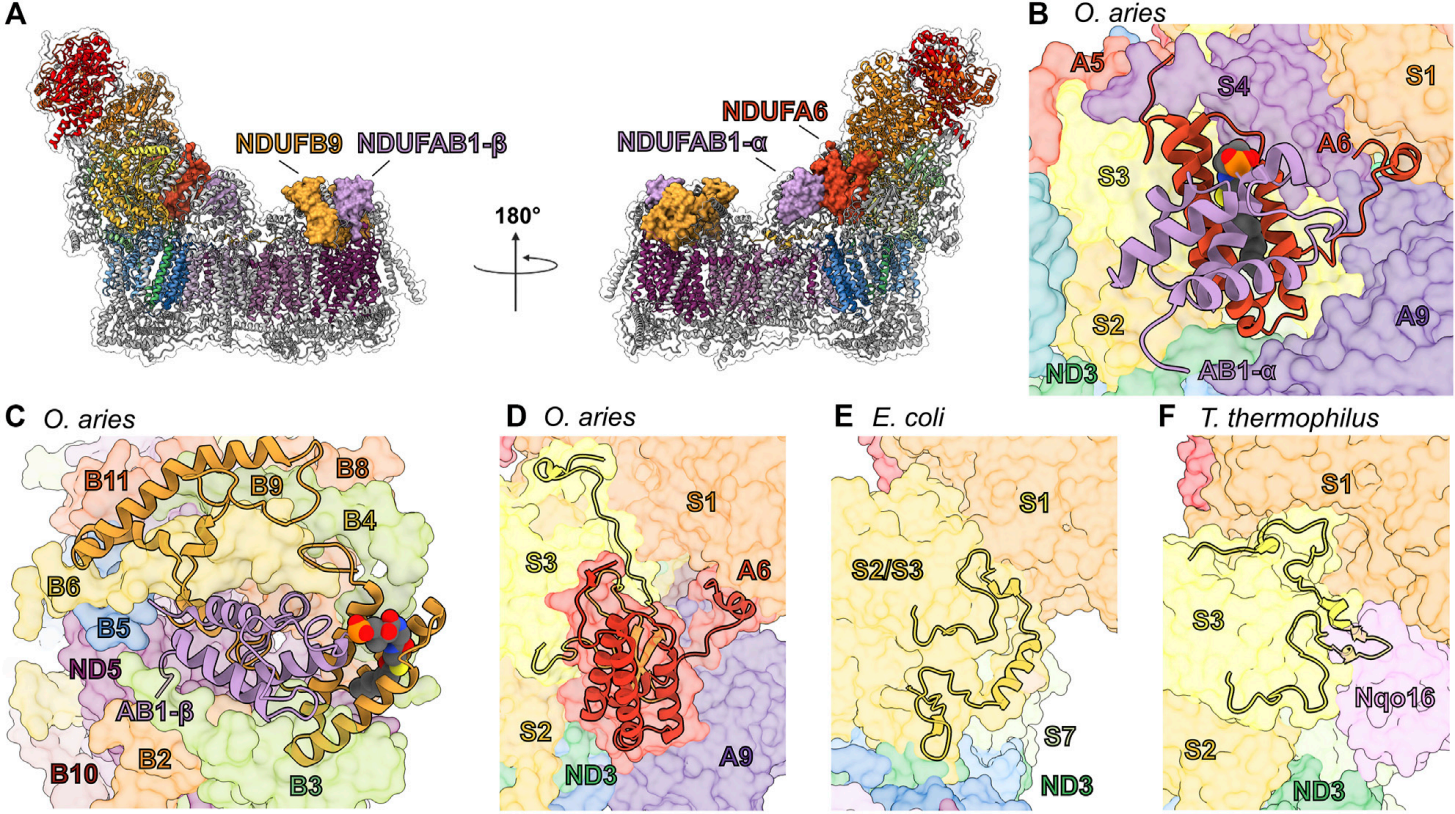
Accessory subunits NDUFA6, NDUFAB1-α, NDUFB9, and NDUFAB1-β in mammalian CI. **(A)** The structure of CI in cartoon with the surfaces of NDUFA6 (cinnabar red)/NDUFAB1-α (violet) and NDUFB9 (yellow orange)/NDUFAB1-β (violet) shown, the core subunits colored as in Figure 1A and the accessory subunits grey. **(B)** Shin view CI showing the NDUFA6/NDUFAB1-β pair in cartoon and other CI subunits as transparent surfaces. The acylated 4′-phosphopantetheine group is shown in spheres (PDB: 5LNK). **(C)** Looking down on the toe of CI (PDB: 6ZKC) showing the NDUFB9/NDUFAB1-β pair in cartoon and the other CI subunits as transparent surfaces. **(D–F)** NDUFA6 replaces structural elements of bacterial CI. Shin view of **(D)** mammalian (PDB: 5LNK), **(E)** *E. coli* (PDB: 7NYR) and **(F)** *T. thermophilus* CI shown as transparent surface with NDUFA6, the NDUFS2/S3 (NuoCD) (corn yellow) linker and NDUFS3 C-termini shown in cartoon. Accessory subunits that are not important for the discussion are omitted from panel **(D)** for clarity. The NDUFS2/S3 (NuoCD) linker in *E. coli* and Nqo16 (pink) subunit in *T. thermophilus* conflict with the position of NDUFA6 in mammals. NDUFA6: cinnabar red, NDUFAB1-α: violet, NDUFB9: yellow orange, NDUFAB1-β: violet/violet, NDUFS2/S3: corn yellow, Nqo16: pink.

The NDUFAB1 subunit of mammalian CI is an acyl carrier protein (ACP) involved in transporting and extending fatty acid chains during fatty acid synthesis. ACPs interact closely with leucine-tyrosine-arginine (LYR) motif proteins (Dibley et al., 2020). The LYR family of proteins are involved with mitoribosome biogenesis (MIEF1-MP) (Brown et al., 2017; Rathore et al., 2018), CII assembly (SDHAF1) (Ghezzi et al., 2009), CIII_2_ assembly (LYRM7) (Maio et al., 2017), and iron-sulfur cluster biogenesis (LYRM4) (Atkinson et al., 2011). These interactions link these different processes to fatty acid biosynthesis and/or are responsible for recruitment of downstream factors (Brown et al., 2017). Mitochondrial CI contains two LYR protein subunits, NDUFA6 and NDUFB9, both of which are found in association with a copy of NDUFAB1 in the intact complex (Figure 6A). Therefore, two copies of NDUFAB1 are present in mitochondrial CI. NDUFAB1-α binds to NDUFA6 on the “shin” side of the Q-module whereas NDUFAB1-β binds to NDUFB9 on the mitochondrial matrix side of the P_D_-module forming part of the P_D_-bulge (Figure 1A and Figure 6A).

NDUFAB1 is a small globular protein composed of four α-helices and a 4′-phosphopantetheine (4PP) group at Ser44 which serves as a prosthetic group for the attachment of fatty acids and which can exist in two conformations: buried, for fatty acid transport; or flipped-out, for interaction with fatty acid modifying enzymes (Cronan, 2014). The interaction between ACPs and LYR proteins *via* the flipped-out 4PP group and acyl chain extending into the core of the LYR protein (Figures 6B,C), was first observed in the structure of ovine CI (Fiedorczuk et al., 2016). The same flipped-out mode of interaction has since been seen for all ACP/LYR protein pairs (Boniecki et al., 2017; Brown et al., 2017). Unlike the case in mammals, *Y. lipolytica* CI does not harbor two identical copies of NDUFAB1 but two distinct homologues, ACMP1 and ACMP2, bound in the analogous positions of NDUFAB1-α and β respectively.

*NDUFA6/NDUFAB1-α*—In fully assembled CI NDUFAB1-α interacts solely with NDUFA6 and joins the complex in the final stages of CI assembly (Figure 2A) (Guerrero-Castillo et al., 2017a). Therefore, NDUFAB1-α is one of only two accessory subunits that does not interact directly with any core subunits (the other being NDUFC1). NDUFA6 is situated in the Q-module interacting with NDUFS3, NDUFS4 and ND3 bridging the peripheral and membrane arms of the complex (Figure 6B). NDUFA6’s C-terminal coil extends toward the N-module and interacts with NDUFS3, NDUFA9 and NDUFS1 (Figure 6B). In *E. coli* the binding site of NDUFA6 conflicts with the loop connecting the fused NDUFS2/NDUFS3 single subunit NuoCD (Figures 6D,E). Whereas, in *T. thermophilus*, the binding site of NDUFA6 conflicts with the accessory subunit Nqo16, which is not present in other species (Figure 6F). Also, in this region differences can be seen between the C-terminal loops of NDUFS3 in mammals and *T. thermophilus* (Nqo5) which have adapted to interact with the distinct accessory subunits NDUFA9 and Nqo16 respectively (Figures 6D,F).

*NDUFB9/NDUFAB1-β*—In contrast to the NDUFA6/NDUFAB1-α pair, NDUFB9/NDUFAB1-β participates early in CI assembly, interacting with core subunit ND5 and accessory subunits NDUFB2, NDUFB3, NDUFB6 and NDUFB8 to form the P_D_-b subassembly (Figure 2 and Figure 6C) (Guerrero-Castillo et al., 2017a). Unlike NDUFA6, no structural elements of the bacterial core subunits conflict with the position of the NDUFB9/NDUFAB1-β pair, suggesting that NDUFB9 simply used the surface of ND5 as a platform without replacing any pre-existing functionality.

*Roles of LYR/ACP pairs in CI assembly and activity*—Given its role in fatty acid metabolism it is not surprising that NDUFAB1 is the only accessory subunit essential for overall HEK293T viability (Stroud et al., 2016). Complementation of NDUFAB1^KO^ HEK293T cells with a yeast ACP rescued cell growth, but not CI assembly (Stroud et al., 2016). This demonstrates that although NDUFAB1 is essential for CI assembly, the essential role of NDUFAB1 in cell viability is independent of CI. NDUFA6^KO^ in HEK293T or NDUFA6 (LYRM6) deletion in *Y. lipolytica* (ΔNDUFA6) results in a subassembly of CI lacking the N-module or a fully assembled (only lacking the NDUFA6/NDUFAB1-α pair), but catalytically incompetent CI, respectively (Stroud et al., 2016; Guerrero-Castillo et al., 2017a). This indicates the NDUFA6/NDUFAB1-α pair is essential for activity (Guerrero-Castillo et al., 2017a). More recently, point mutations of NDUFA6, generated in *Y. lipolytica*, at its interface with core membrane subunits ND1 and ND3 (Figure 6B) demonstrated that NDUFA6’s essential role in CI activity is mediated by contacts at this site (Angerer et al., 2014) (Galemou Yoga et al., 2020). Structural analysis of the functionally impaired F89A^A6(LYRM6)^ mutant revealed that this mutation influences the structures of the TMH1-2^ND3^ loop, TMH5-6^ND1^ loop, and NDUFA9; this network of loops is proposed to provide needed conformational flexibility during ubiquinone reduction (Galemou Yoga et al., 2020). Conversely, NDUFB9^KO^ in HEK293T cells more closely recapitulates, though with lesser severity, the NDUFAB1^KO^ phenotype in which the assembly of CI is abrogated (Stroud et al., 2016). This demonstrates the importance of the NDUFB9/NDUFAB1-β pair in the early stages of CI assembly.

The above indicates that CI assembly and activity are dependent on the presence of acylated ACPs and leads to the hypothesis that the LYR/ACP pairs connect CI assembly and activity to fatty acid metabolism. The NDUFB9/NDUFAB1-β and NDUFA6/NDUFAB1-α pairs ensures that new CI is not assembled, and that CI activity is “switched off” in the absence of sufficient levels of fatty acid fuel, i.e., low levels of acylated ACP. In metazoans that rely solely on respiration for energy production, the dependence of CI on the presence of sufficient fatty acids is likely moot, as the organism would be unlikely survive such severe starvation conditions. However, during the evolution of eukaryotes dependence of CI assembly and activity on the presence of sufficient levels of fatty acid may have aided in switching between different metabolic strategies. The fact that *Y. lipolytica* CI harbors two distinct ACP homologues, ACMP1 and ACMP2, may provide additional flexibility in regulating CI assembly vs. activity. This combined with its powerful genetic tools makes *Y. lipolytica* a powerful system in which to test the distinct roles of the ACPs in CI assembly and activity.

### The NADPH Containing Subunit NDUFA9

**FIGURE 7:**
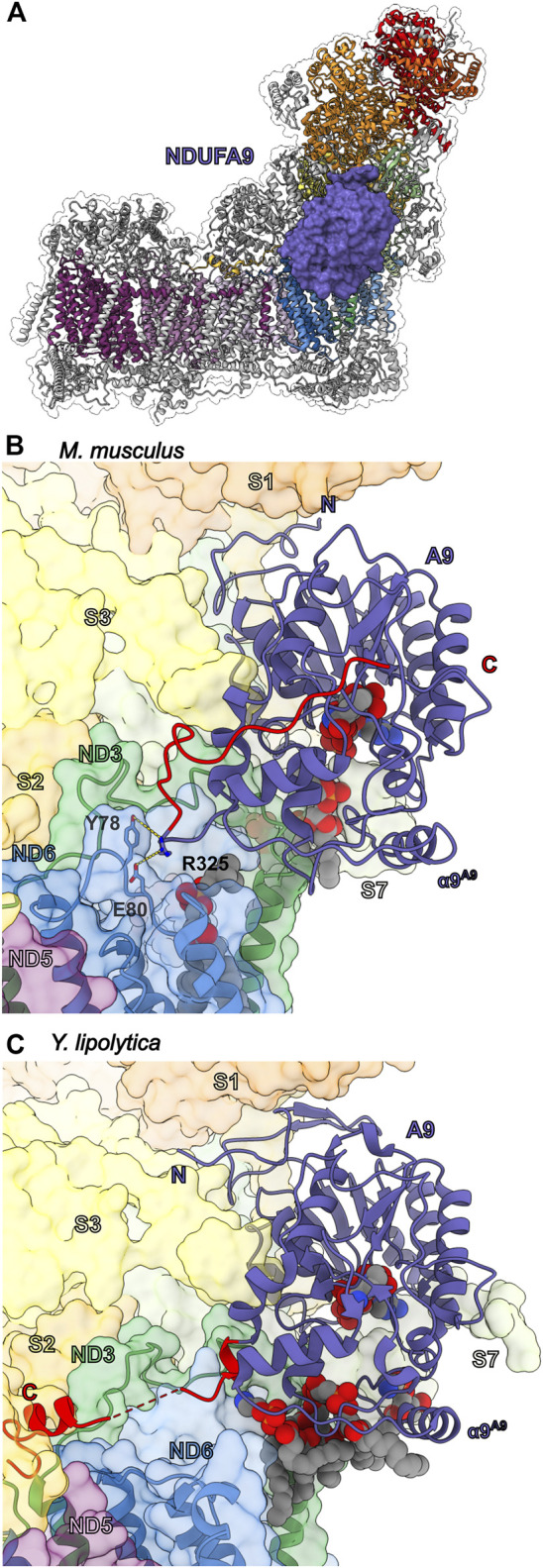
Accessory subunit NDUFA9. **(A)** The structure of CI (PDB: 6ZKC) in cartoon with the surface of NDUFA9 (lavender) shown, the core subunits colored as in Figure 1A and the accessory subunits grey. **(B)** *M. musculus* CI (PDB: 6G2J) and **(C)** *Y. lipolytica* CI (PDB: 6RFR) shown as transparent surfaces with NDUFA9 (NUEM), ND3 (sea green) and ND6 (cyan-blue) shown in cartoon. NADPH and lipids are shown as spheres. The C-terminal coils of NDUFA9 are colored red. The key residues interacting with ND6 are shown in stick representation. Accessory subunits that are not important for the discussion are omitted from the figures for clarity. Subunits are colored as in Figure 1 in all the structure figures unless stated otherwise. NDUFA9: lavender, ND3: sea green, ND6: cyan-blue.

NDUFA9 is part of the Q-module adjacent to the membrane at the Q/P interface (Figure 1B and Figure 7A). Along with NDUFA1 and assembly factor NDUFAF2, NDUFA9 is incorporated after formation of the Q/P_P_ subassembly (Figure 2A) (Sánchez-Caballero et al., 2016; Guerrero-Castillo et al., 2017a). NDUFA9 belongs to the family of short-chain dehydrogenase/reductases and contains a conserved nucleotide binding Rossmann fold motif that binds a NADPH cofactor (Figure 7B). NDUFA9 forms an extensive interface with the core-subunits NDUFS1, NDUFS3, NDUFS7 and NDUFS8, as well as ND1, ND3 and ND6 (Figure 7B). NDUFA9 binds on the surface of membrane overhanging the opening of CoQ tunnel in ND1 (Figure 3L). The short amphipathic helix of NDUFA9 (α9^A9^) along with those of NDUFS7 (α1^S7^ and α6^S7^) and NDUFA12 (α2^A12^) pull the membrane lipids in the region out of the membrane plane by ∼10 Å (Figure 3L) (Parey et al., 2019). Additionally, the C-terminal amphipathic helix of NDUFS7 (α6^S7^) approaches NDUFA9’s NADPH binding pocket and may contact the NADPH directly (Fiedorczuk et al., 2016). Distortion of the membrane at the entry site of the hydrophobic CoQ substrate has been suggested to help promote CoQ entry and exit from the CoQ tunnel (Parey et al., 2019).

Despite its homology to dehydrogenase enzymes, NDUFA9 has not be shown to have any catalytic activity and the bound NADPH cofactor most likely plays a structural role as demonstrated by mutations that block NADPH binding in *Y. lipolytica* only affecting CI assembly but not activity (Abdrakhmanova et al., 2006; Ciano et al., 2013; Torraco et al., 2017). Deletion of NDUFA9 in *Y. lipolytica* (NUEM) or *Neurospora crassa* (Nuo-40) abolished CI activity and severely decreased the total amount of CI reflecting a severe assembly defect (Schulte et al., 1999; Abdrakhmanova et al., 2006). In HEK293T cells, NDUFA9^KO^ results in loss of CI activity and the appearance of a novel ∼600-kDa subassembly lacking the N-module as well as components of the Q-module (Stroud et al., 2013). NDUFA9 missense mutations R321P^A9^ and R360C^A9^ are associated with human mitochondrial disease (van den Bosch et al., 2012; Baertling et al., 2018). R321^A9^ is in the core of the Rossmann fold and forms a salt bridge with Asp/Glu133^A9^ (Asp in humans, Glu in other mammals) adjacent to the NADPH binding site. Therefore, like the NADPH disrupting mutations, mutation of R321^A9^ to proline likely disrupts the stability of NDUFA9 and its interaction with CI. However, R360^A9^ (R325 in *M. musculus*) is not located in the core of the protein but on the C-terminal loop where it packs against Tyr78^ND6^ of the TMH3-4^ND6^ loop, suggesting an important role for the interaction between NDUFA9 and the core TM subunits (Figure 7B).

Cross-linking studies have shown that in the deactive (D)-form of CI, ND3 crosslinked to NDUFA9 but not in the active (A)-form (Ciano et al., 2013). The order to disorder transitions seen in the TMH1-2^ND3^ loop and the TMH 3-4^ND6^ loop between the A- and D-forms of the enzyme are also reflected in a structurally conserved short amphipathic helix of NDUFA9 (human α9^A9^), which is ordered in the A-form but disordered in the D-form (Kampjut and Sazanov, 2020). In *Y. lipolytica* mutations in NDUFA6 (LYR6M) adjacent to the TMH1-2^ND3^ loop (F89A^A6(LYRM6)^) result in disorder of the Q-site loops of the core subunits (the TMH1-2^ND3^, TMH5-6^ND1^ and β1-β2^S2^ loops), as well as the C-terminal loop of NDUFA9 propagating over 50 Å away from the mutation site (Galemou Yoga et al., 2020). The disorder of NDUFA9 also results in the loss of density for several NDUFA9 associated lipid molecules adjacent to the CoQ tunnel entry (Galemou Yoga et al., 2020).

Importantly, the above demonstrates that the interactions between NDUFA9 and the core TM subunits are key to CI activity in both mammals and yeast. However, significant structural differences exist in the interactions of the NDUFA9 C-terminus in mammals and yeast (Figures 7B,C). In mammals, the C-terminus of NDUFA9 interacts closely with both ND3 and ND6 but then folds back onto the core of the Rossman fold (Figure 7B). Conversely, in *Y. lipolytica*, the NDUFA9 C-terminus extends across the surface of the membrane arm and buries an α-helix adjacent to ND4L and the NDUFS2 N-terminal coil (Figure 7C). Despite these differences the hypothesis emerges that NDUFA9, due to its close interaction with the Q-site loops and role in distorting the membrane around the Q-tunnel, may help regulate substrate access to the CoQ-tunnel by altering the membrane environment in response to changes in the conformation of the Q-site loops. The order to disorder transitions of the NDUFA9 C-terminus, seen in the A- and D-forms of the mammalian enzyme and in the *Y. lipolytica* NDUFA6 mutant, propagate to the membrane and likely disrupt the lipid environment generated by NDUFA9, NDUFS7 and NDUFA12 (Figure 3L). Therefore, NDUFA9 may regulate access to the CoQ site in the A/D transitions and during early stages of CI assembly.

### Transmembrane Accessory Subunits of the Heel: NDUFA13, NDUFA3 and NDUFA1

**FIGURE 8:**
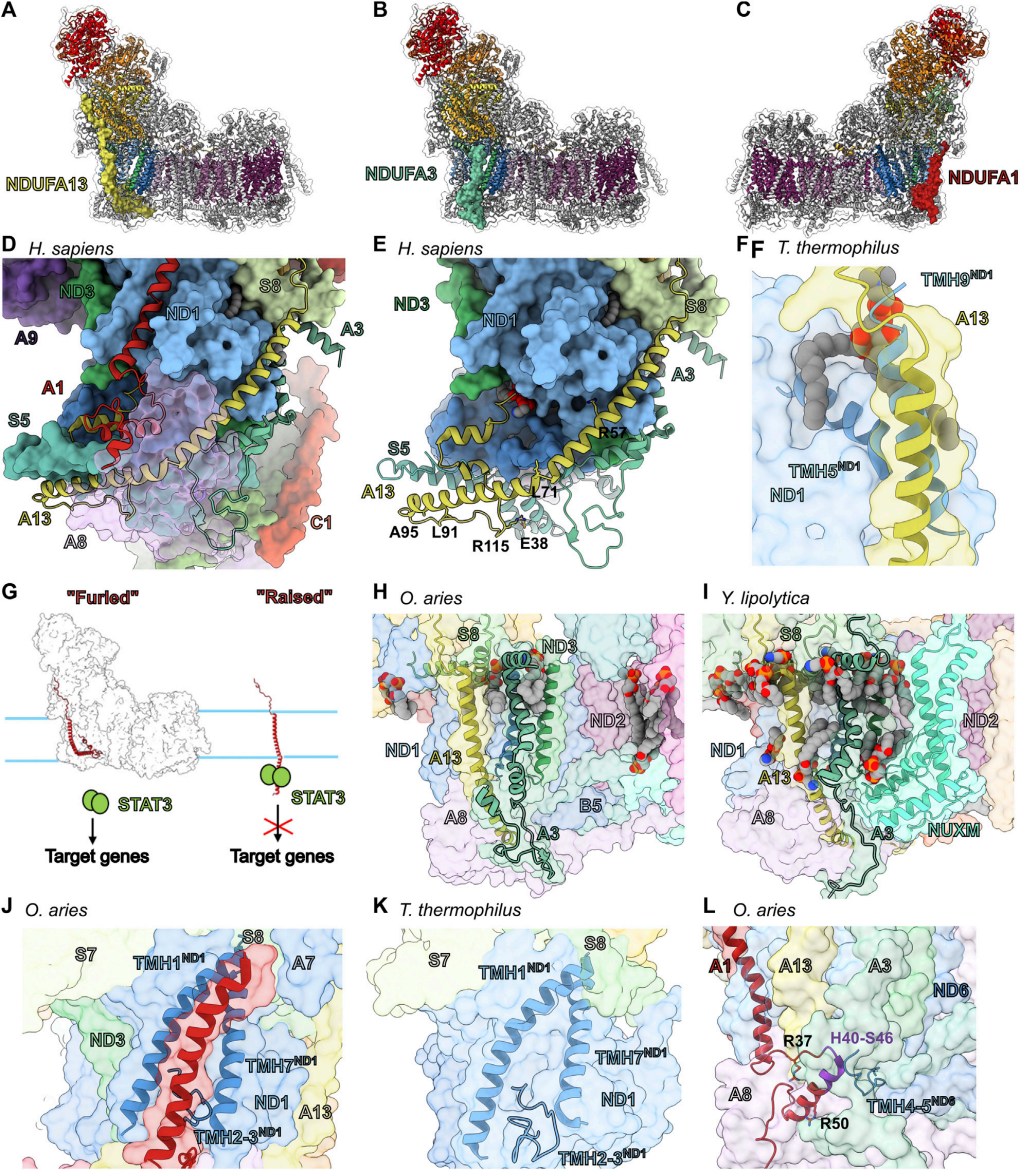
Accessory subunits of the CI heel. The structure of CI (PDB: 6ZKC used throughout) in cartoon with the surface of NDUFA13 (light yellow) **(A)**, NDUFA3 (pine green) (**B)** and NDUFA1 (firebrick red) **(C)** shown, the core subunits colored as in Figure 1A and the accessory subunits grey. **(D)** Looking up from the CI heel showing NDUFA13, NDUFA3, and NDUFA1 of *H. sapiens* CI structure (PDB: 5XTD) as cartoons. NDUFA8 (lilac purple) is shown as a transparent surface for clarity. **(E)** Accessory subunit NDUFA13 of *H. sapiens* CI structure (PDB: 5XTD) is shown as cartoon. The residues mentioned in the text are shown in stick representation. **(F)** NDUFA13 compensates for the lost transmembrane helix TMH9^ND1^ (cobalt blue) of *T. thermophilus*. *T. thermophilus* ND1 (Nqo8) (PDB: 4HEA used throughout) is superposed on ND1 of mammalian (*O. aries*) CI structure shown as transparent surface. TMH5^ND1(Nqo8)^ and TMH9 ^ND1(Nqo8)^ are shown as cartoons. NDUFA13 surface and cartoon are shown. **(G)** Schematic representation of the “Flag post model.” NDUFA13 of healthy CI is furled and incapable of binding to STAT3. NDUFA13 alone is raised exposing the STAT3 interacting site to the IMS. **(H,I)** Assembly vs. lipid binding roles of NDUFA3. **(H)** Mammalian CI shown as transparent surface with NDUFA3, NDUFA13, α1^ND1^ (cobalt blue), and α3^ND3^ (sea green) shown as cartoons. **(I)** *Y. lipolytica* CI (PDB: 6YJ4) shown as transparent surface with NDUFA3 (NI9M), NDUFA13 (NB6M), NUXM (light cyan), α1^ND1^ (NU1M), and α3^ND3^ (NU3M) (sea green) shown as cartoons. **(J,K)** NDUFA1 compensates for the shorter TMH2-3^ND1^ loop. **(J)** Mammalian CI structure shown as transparent surface with NDUFA1, TMH1^ND1^, TMH7^ND1^ and the TMH2-3^ND1^ loop shown as cartoons. **(K)** *T. thermophilus* CI shown as transparent surface with TMH1^ND1(Nqo8)^, TMH7^ND1(Nqo8)^ and the TMH2-3^ND1(Nqo8)^ loop shown as cartoon. **(L)** NDUFA1 connects the P_P_-a and P_P_-b subassemblies. Mammalian CI a transparent surface with NDUFA1 and the ND6 TMH4-5^ND6^ (cyan-blue) loop shown as cartoon. Residues mentioned in the text are shown as sticks. Key H40-S46 sequence shown in purple. Subunits are colored as in Figure 1 in all panels unless stated otherwise. NDUFA13: light yellow, NDUFA3: pine, NDUFA1: firebrick red, NDUFA8: lilac purple, ND1: cobalt blue, ND3: sea green, ND6: cyan-blue, NUXM (light cyan).

The heel of CI is the site of CoQ reduction. This region is composed of the core subunits ND1, which harbors the entry site of the CoQ tunnel, three accessory transmembrane subunits NDUFA13, NDUFA3 and NDUFA1 (Figures 8A–C), as well as the quadruple-CX_9_C motif containing subunit NDUFA8 (discussed below) (Figure 8D).

*NDUFA13*—Along with ND1, NDUFA3 and NDUFA8, NDUFA13 forms the P_P_-a subassembly (Figure 2). NDUFA13 has an N-terminal coil that binds along the Q-module, a single TMH that extends into the intermembrane space (IMS) followed by a coil and two short α-helices at the C-terminus in a helix-turn-helix motif (Figures 8A,E). The N-terminal coil of NDUFA13 binds atop the ascending coil of NDUFA7 near the interface of the N- and Q-modules (Figure 8A) and follows a groove along NDUFS2 towards the membrane (Figure 5I). At the interface of the matrix and IMM, NDUFA13 interacts with NDUFS8 and a lipid molecule (Figure 8D). The NDUFA13 TMH binds to ND1 in the membrane and continues into the IMS where it interacts with ND3, NDUFA3 and NDUFA8 (Figure 8D). Although not completely vertical in the membrane the interaction with NDUFA8 bends the NDUFA13 TMH sharply, making it run nearly parallel to the surface of the membrane (Figures 8D,E). Thus, the NDUFA13 TMH extends across the IMS side of the complex interacting with ND6, NDUFB5, NDUFA1, NDUFS5 and NDUFA8 (Figures 8D,E). After crossing the complex, NDUFA13 turns back interacting with NDUFS5, NDUFA8, NDUFB5, ND6, ND3 and NDUFA1 (Figures 8D,E). The NDUFA13 C-terminal coil also participates in the binding of a lipid molecule between ND1, ND3 and ND6 (Figure 8E). In *T. thermophilus* CI, ND1 (Nqo8) has an additional C-terminal TMH (TMH9^ND1(Nqo8)^) that occupies the same position as the NDUFA13 TMH in eukaryotes (Figure 8F), however, this additional TMH is not seen in the *E. coli* CI structure (Baradaran et al., 2013; Kolata and Efremov, 2021). Nonetheless, the presence of the additional helix in *T. thermophilus* ND1 suggests that the position of NDUFA13 binding at the N-terminus of the tilted TMH5^ND1^ may confer additional stability to ND1 (Figure 8F).

In HEK293T cells, NDUFA13^KO^ results in loss of the N-module, NDUFA10 and NDUFB5 indicating that, although it is added early in assembly, NDUFA13 is necessary for the final stages of CI assembly (Stroud et al., 2016). NDUFA13 mutations K5N^A13^ and R115P^A13^ have been implicated in Oxyphil or Hurthle cell tumors (Máximo et al., 2005). The side chain of K5^A13^ is solvent exposed and does not make any specific interaction with other residues, hence mutation of this residue is unlikely to affect the structural integrity of CI directly but may affect mitochondrial targeting and import. R115^A13^ is on the TMH1-α1^A13^ loop and forms a salt bridge with NDUFS5 conserved residue E38^S5^ (Figure 8E). This salt bridge likely helps to stabilize the kinked structure of the NDUFA13 TMH and the R115P^A13^ mutation would remove this stabilizing interaction. Germline mutation R57H^A13^ leads to early onset of hypotonia, dyskinesia and sensorial deficiencies (Angebault et al., 2015). R57^A13^ is buried in a pocket at the interface with ND1 (Figure 8E) and mutation would disrupt this interaction and weaken the association of NDUFA13 with CI.

NDUFA13 is also known as GRIM-19 and was independently discovered as part of an apoptosis/cell proliferation pathway involving the cytoplasmic transcription factor STAT3 (Lufei et al., 2003). Through its interaction with STAT3, NDUFA13 represses STAT3 dependent transcription and thus has an anti-proliferative pro-apoptotic effect and plays a role in tumor suppression (Lufei et al., 2003). The interaction between NDUFA13 and STAT3 was narrowed down to the DNA binding domain and linker region of STAT3 and residues 36–72 of NDUFA13 (Lufei et al., 2003), however, given that residues 29–51 of NDUFA13 are buried in the membrane, the most likely interaction site would be between residues 52–72 which extend into the IMS. Consistent with this, mutations in the IMS portion of the NDUFA13 TMH (L71P, L91P and A95T in human) disrupt STAT3 binding and promote oncogenesis (Nallar et al., 2013). When associated with CI, this region of NDUFA13 (residues 52–72) is inaccessible due to interactions with NDUFA8 and NDUFA3 (Figure 8D). Hence, NDUFA13 can only interact with STAT3 when it is not bound to CI. These observations lead to a “flagpole” hypothesis in which NDUFA13 acts as a sensor that ties mitochondrial and ETC health to cell proliferation and apoptosis (Figure 8G). In conditions that block CI assembly or promote CI disassembly the NDUFA13 “flag” is raised allowing it to interact with STAT3 suppressing proliferation and promoting apoptosis (Figure 8G). Conversely, in healthy mitochondria, NDUFA13 is “furled,” i.e., interacting with CI, and thus sequestered away from interaction with STAT3 thereby promoting proliferation (Figure 8G).

*NDUFA3*—Along with NDUFA13, NDUFA8 and ND1, NDUFA3 is a member of the P_P_-a subassembly (Figure 2A) (Sánchez-Caballero et al., 2016). NDUFA3 is made up of an amphipathic helix that lies at the matrix-membrane interface, a single TMH, and an α-helix followed by a C-terminal coil in the IMS (Figures 8B,H). The NDUFA3 amphipathic helix interacts with the N-terminal NDUFS8 amphipathic helix and helps to trap two lipids, one against ND1 and the other against ND3 (Figure 8H). In the membrane and IMS, NDUFA3 interacts with ND3 and ND1 *via* a short α-helix and NDUFA13, ND6, NDUFS5, NDUFB5 and NDUFA8 *via* a C-terminal coil. Thus, NDUFA3’s interactions bridge components of the Q, P_P_-a and P_P_-b subassemblies within the matrix and IMS (Figure 8H).

In HEK293T cells, NDUFA3^KO^ blocks CI assembly and leads to a decrease in the level of subunits in the N- and P_P_-b subassemblies, as well as subunits in the P_D_-a subassembly (Stroud et al., 2016). NDUFA3 knockdown in human cell lines also showed that NDUFA3 is required for the assembly and stability of the Q-module (Rak and Rustin, 2014). In the structure of *Y. lipolytica* CI (Grba and Hirst, 2020) the amphipathic helix of NDUFA3 (NI9M), along with subunit NUXM, trap several lipids against ND3 and ND2 (Figure 8I). However, in mammals the loss of the first three TMHs of ND2, which form the binding site of NUXM, results in the lack of a homolog for NUXM and fewer structured lipids in this region (Figure 8H). Interestingly, a NUXM homologue is also seen in plants (coined NDUX1) emphasizing this metazoan specific deletion in ND2 (Maldonado et al., 2020).

This leads to the hypothesis that one of the original functions of NDUFA3 was to stabilize lipid binding at the interface of ND1, ND3 and ND2, however with the truncation of ND2 and loss of NUXM, this function may be minimized in metazoans. The importance of NDUFA3 in CI assembly likely stems from interactions at two interfaces. 1) At the interface of the matrix and IMM, the NDUFA3 amphipathic helix, along with NDUFA13, sandwich the N-terminal amphipathic helix of NDUFS8, anchoring the Q subassembly to the Pp-a subassembly (Figure 8H). 2) In the IMS NDUFA3’s helix binds between ND1 and ND3, thereby stabilizing the association of the Pp-a and Pp-b subassemblies. This interaction involving ND1 and ND3 is much diminished in *Y.lipolytica* CI where NDUFA3 (NI9M) lacks the IMS α-helix but binds several additional lipids not seen in mammalian structures (Figure 8I), suggesting the possibility of different primary roles, lipid biding vs. assembly, in different organisms.

*NDUFA1*—The single TMH accessory subunit NDUFA1 is not found in the P_P_-a or P_P_-a/Q subassemblies but joins along with NDUFA9 upon connection of the P_P_-a/Q and P_P_-b subassemblies forming the Q/P_P_ or Q/P intermediates (Figure 2A) (Sánchez-Caballero et al., 2016). NDUFA1 does not have an N-terminal coil but begins at the interface of the matrix and IMM with its TMH (Figures 8C,J). In the IMS, NDUFA1 has a small domain comprised of a loop, α-helix (α1^A1^) and C-terminal coil. At the interface of the matrix and IMM, the NDUFA1 N-terminus interacts with NDUFS8 and NDUFA7 (Figure 8J). Also, at the matrix/IMM interface the amphipathic helix of NDUFA12 binds atop NDUFA1 trapping a cardiolipin onto the NDUFA1 TMH (Figure 3L). In the membrane, NDUFA1 interacts with ND1 filling a groove formed between TMH1^ND1^ and TMH7^ND1^ (Figure 8J). In the IMS, NDUFA1 interacts with NDUFA8, ND1, ND6, NDUFS5 and NDUFA13. In *T. thermophilus* the ND1 (Nqo8) groove that houses NDUFA1 in eukaryotes is partially filled by the TMH2-3^ND1(Nqo8)^ loop which is 10 residues longer relative to that seen in eukaryotes (Figure 8K). In the *E. coli* CI structure, TMH1^ND1(NuoH)^ is disordered and the TMH2-3^ND1(NuoH)^ loop is of intermediate length (5 amino acid residues longer than the eukaryotic loop), suggesting that the TMH2-3^ND1^ loop is important for the stability of TMH1^ND1^.

NDUFA1 has been shown to play an essential role in the assembly pathway and function of CI in mammals (Fernandez-Moreira et al., 2007). NDUFA1^KO^ in HEK293T cells prevents the full assembly of the complex and leads to a drop in the levels of subunits associated with the N- and P_P_-b subassemblies (Stroud et al., 2016). The promoter region of the *NDUFA1* gene contains a cAMP response element suggesting it is linked to cAMP signaling pathways that regulate cellular energy metabolism (Palmisano et al., 2007). Studies in Chinese hamster cells identified several important functional residues and regions of NDUFA1 (Breen and Scheffler, 1979; Au et al., 1999; Yadava et al., 2002). For example, the conservative mutation R50K^A1^ results in a severe loss of CI activity (Yadava et al., 2002). The structure shows that R50^A1^ forms an inter-subunit salt bridge with E77^A13^ buried in an otherwise relatively hydrophobic pocket (Figure 8L). The inability of the R50K^A1^ to maintain this interaction speaks to the specificity and importance of the interaction between NDUFA1 and NDUFA13. Furthermore, sequence differences between rodent and primate NDUFA1 in the region from H40-S46^A1^ in humans was also shown to prevent complementation between the NDUFA1 sequences from the different species (Yadava et al., 2002). Swapping just a few residues between the hamster and the human sequence results in CI assembly defects (Yadava et al., 2002). This region of the NDUFA1 interacts most closely with ND6 highlighting the importance of this interaction for CI assembly (Figure 8L). Additionally, mutations of highly conserved residues (G8R^A1^ and R37S^A1^) were found in two patients with Leigh’s syndrome and with myoclonic epilepsy and developmental delay (Fernandez-Moreira et al., 2007). G8^A1^ lies within the first 28 amino acids required for mitochondrial targeting, import, and orientation of NDUFA1 but also packs tightly against ND1 indicating either an import defect or assembly defect due to impaired interaction of NDUFA1 and ND1 (Fernandez-Moreira et al., 2007). R37^A1^ forms inter-subunit salt bridges with D89^A8^ and E94^A8^ of NDUFA8 (Figure 8L).

The above leads to the hypothesis that NDUFA1 through its interaction with ND1, ND6, NDUFA8 and NDUFA13, is required for the stable connection of the P_P_-a and P_P_-b subassemblies during CI biogenesis. Given that none of the Q/P_P_-a subassembly subunits are directly blocking NDUFA1 binding, it is unclear from the structure of the intact complex what prevents association of NDUFA1 to the Q/P_P_-a subassembly. This suggests a conformation change upon association of the P_P_-b subassembly that would expose the NDUFA1 binding site. Notably, TMH1^ND1^, which is flexible in the *E. coli* structure (Kolata and Efremov, 2021), interacts closely with ND3 which arrives as part of the P_P_-b subassembly. Altogether, this suggests that NDUFA1 compensates for the shortened TMH2-3^ND1^ loop as a “wedge” that stabilizes the position of TMH1^ND1^ and whose binding site is not fully available until the position of TMH1^ND1^ is established through interaction with ND3. Thus, NDUFA1 would only bind after association of the Q/P_P_-a and P_P_-b subassemblies and further stabilizes their association *via* a network of interactions (Figure 8L). Furthermore, the addition of NDUFA12 in the final stages of CI biogenesis, which binds overtop of NDUFA1 at the interface of the matrix and IMM, would act to lock NDUFA1 in place further stabilizing its association.

### The Nucleoside Kinase Subunit: NDUFA10

**FIGURE 9:**
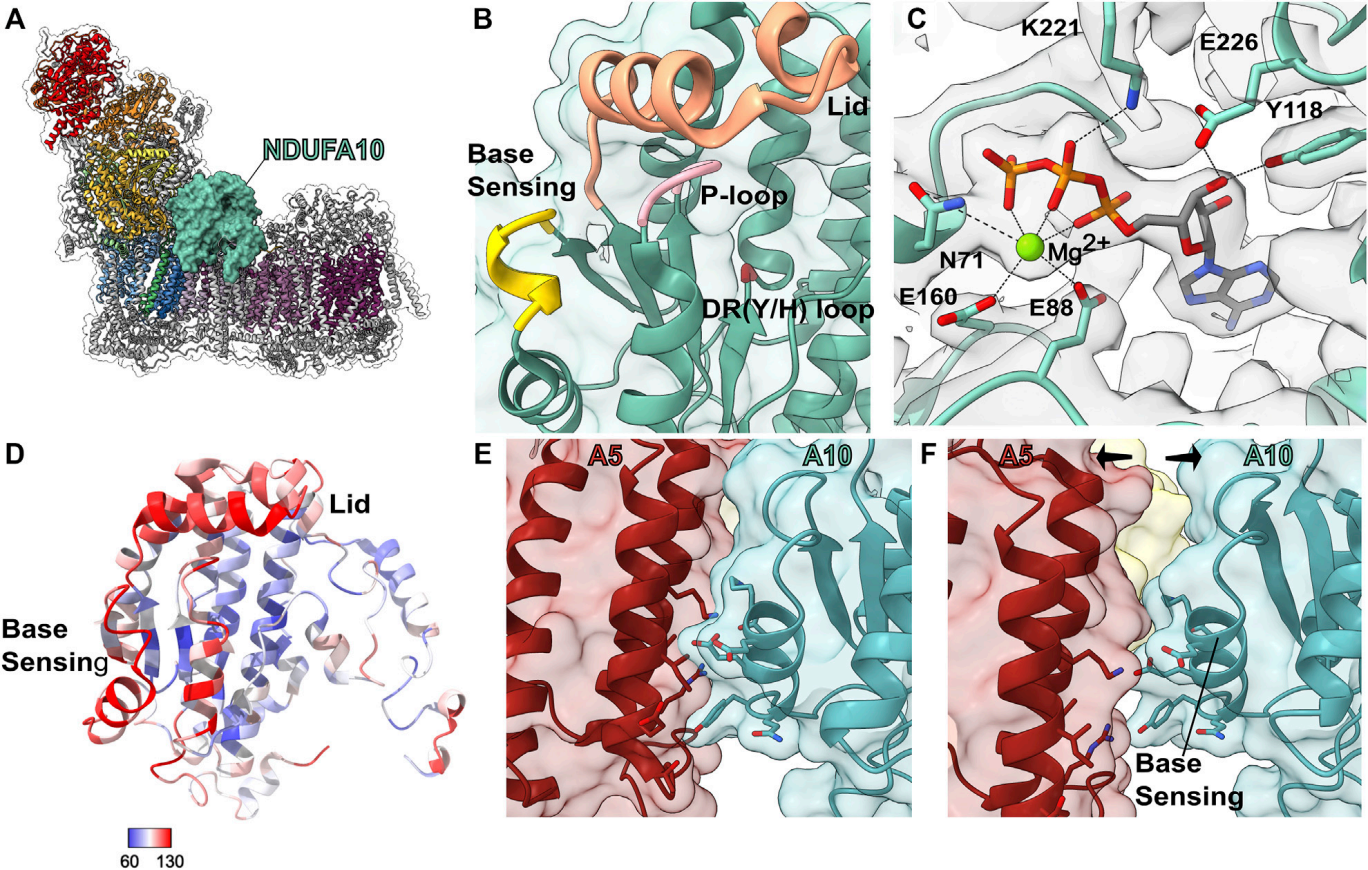
Accessory subunit NDUFA10. **(A)** The structure of CI (PDB: 6ZKC) in cartoon with the surface of NDUFA10 (cadet blue) shown, the core subunits colored as in Figure 1A and the accessory subunits grey. **(B)** Transparent surface and cartoon of *O. aries* NDUFA10 (PDB: 6ZKC). The P-loop (pink), DR (Y/H) motif (red), lid region (orange) and base sensing loop (yellow) are shown as thickened cartoon. **(C)** Cartoon of NDUFA10 with the density map (grey) from *M. musculus* CI bound to the Q-site inhibitor piericidin (PDB: 7B93). Mg^2+^ ATP and key binding residues are shown as sticks and colored by element. **(D)** Cartoon of NDUFA10 colored by atomic displacement parameter (ADP) in CI (PDB: 6ZKO). Red: most flexible, Blue: least flexible. **(E,F)** State dependent interactions between NDUFA5 and NDUFA10 involve the base-sensing loop. **(E)** Transparent surface of closed A-state CI showing NDUFA10 and NDUFA5 (auburn) as cartoons (PDB: 6ZKC). **(F)** Transparent surface of open D-state CI showing NDUFA10 and NDUFA5 as cartoons (PDB: 6ZKD). Key residues involved in the interaction are shown as sticks and colored by element. NDUFA10: cadet blue, NDUFA5: auburn.

NDUFA10 is a globular protein of the nucleoside kinase family (Steeg et al., 2011) that lies on the matrix side of ND2 (Figure 9A). It is one of the last assembled subunits of the P_P_-b subassembly joining along with NDUFS5, NDUFB4 and the P_D_-a subassembly to make either the P_P_-b/P_D_-a or Q/P subassemblies (Figure 2A) (Sánchez-Caballero et al., 2016). Addition of NDUFA10 as a subunit of CI occurred in metazoans and no structural homolog for NDUFA10 is seen in fungi or plants (Elurbe and Huynen, 2016). NDUFA10 interacts with accessory subunits NDUFC1, NDUFA5, NDUFB11 and NDUFC2 and core subunits ND2 and NDUFS2. The N-terminal coil of NDUFA10 occupies space at the interface of the matrix and IMM that is occupied by the first three ND2 TMH in bacteria, fungi, and plants. In HEK293T cells, NDUFA10^KO^ leads to the loss of the N-module as well as NDUFB4 which is added to the complex in the same step as NDUFA10 (Figure 2A) (Stroud et al., 2016). This suggests that in the absence of NDUFA10 the assembly of the complex stalls at the Q/P intermediate.

NDUFA10 is related to deoxyguanosine (dG) kinases, which catalyze phospho-transfer reactions between a donor ATP and a dG acceptor, generating ADP and dGMP. Theses enzymes have two substrate binding sites, a deep pocket for binding the acceptor nucleoside and a more exposed binding site for the donor ATP nucleotide (Ostermann et al., 2000; Sabini et al., 2008). However, it has also been shown that ATP nucleotide can bind to the acceptor nucleoside pocket acting as a feedback inhibitor of the phospho-transfer reaction (Welin et al., 2007). Four loops are important for substrate binding and catalysis in dG kinases: the P-loop (β1-α1^A10^ loop), the DR(Y/H) motif containing loop (^160^ERS^162^ in human NDUFA10); the “lid” region (D173-I194^A10^); and the base-sensing loop that recognizes the donor nucleotide base (S224-E229) (Figure 9B) (Ostermann et al., 2000; Sabini et al., 2008). It is known that in dG and related nucleoside kinases the lid region and base-sensing loops undergo conformational change upon binding of substrate (Vonrhein et al., 1995; Sabini et al., 2008).

As has been noted previously (Elurbe and Huynen, 2016), many of the residues for binding nucleoside in the acceptor pocket are conserved in NDUFA10. Although the quality of density for bound substrate varies significantly, mammalian CI structures have been modeled either empty or with ATP, ADP or AMP in the NDUFA10 acceptor binding pocket (Fiedorczuk et al., 2016; Wu et al., 2016; Guo et al., 2017; Agip et al., 2018; Bridges et al., 2020; Kampjut and Sazanov, 2020; Chung et al., 2021; Yin et al., 2021). Most CI density maps obtained thus far have been at 3–4 Å resolution and this medium resolution can lead to difficulty in the modeling and interpretation of bound ligands. Therefore, when analyzing the presence of bound ligands at medium resolution it is important to examine cryoEM maps directly. The cryoEM map with the clearest density for the bound substrate is the mouse CI bound to the Q-site inhibitor Piericidin A at 3.0 Å (Bridges et al., 2020). Although substrate bound to NDUFA10 is modeled as ATP, the density is most consistent with Mg^2+^ ATP or potentially Mg^2+^ deoxyATP (dATP) (Figure 9C). The Mg^2+^ ion is coordinated by E88^A10^ and E160^A10^. E160^A10^ is part of the DR(Y/H) motif loop and the equivalent residue in thymidylate kinase has also been shown to coordinate a Mg^2+^ ion (Whittingham et al., 2010). In the different CI structures solved to date the lid region and base-sensing loop are more flexible, with weaker cryoEM density and higher atomic displacement parameters (ADPs) than the other regions of NDUFA10 (Figure 9D). The clearest cryoEM density for these loops appears in the mouse structures with nucleotide bound (Bridges et al., 2020), suggesting a role for substrate in stabilizing these loops, *via* interactions between K221^A10^ and D191^A10^ of the lid region and the β-phosphate and 3′-hydroxyl of the bound nucleotide, respectively (Figure 9C), similar to what is seen in other nucleoside kinases (Vonrhein et al., 1995; Sabini et al., 2008). The ‘base-sensing’ loop of NDUFA10 (mainly via α9^A10^) participates in a state-dependent interaction with NDUFA5 (Figures 9E,F). The peripheral arm of CI rotates between the D-state and the A-state and brings NDUFA5 into contact with the base-sensing loop of NDUFA10 only in the A-state (Figure 9E).

The above leads to the hypothesis that NDUFA10 is a nucleotide receptor that may influence the A-to-D state transition. Flexibility in the lid and base-sensing loops may impose an entropic barrier weakening the interaction between NDUFA10 and NDUFA5. If so, binding of nucleotide in the acceptor site may remove that barrier and hence help to promote the active state of the complex. Conversely, as has been proposed previously from cryoEM map local resolution analysis (Letts et al., 2019), flexibility within NDUFA10 may allow for conformational changes to be transmitted from the peripheral arm into the membrane arm. In this scenario, nucleotide binding may limit conformational coupling across NDUFA10. In either case, evolution would have transformed an enzyme that recognizes nucleotide substrate through a series of conformational changes into a nucleotide sensor that influences CI activity. This hypothesis generates several experimentally testable predictions, the major one being that the CI activity or A-to-D transition would be sensitive to nucleotide concentration.

### Transmembrane Subunits at the Interface of ND2 and ND4: NDUFC2, NDUFC1 and NDUFB1

**FIGURE 10:**
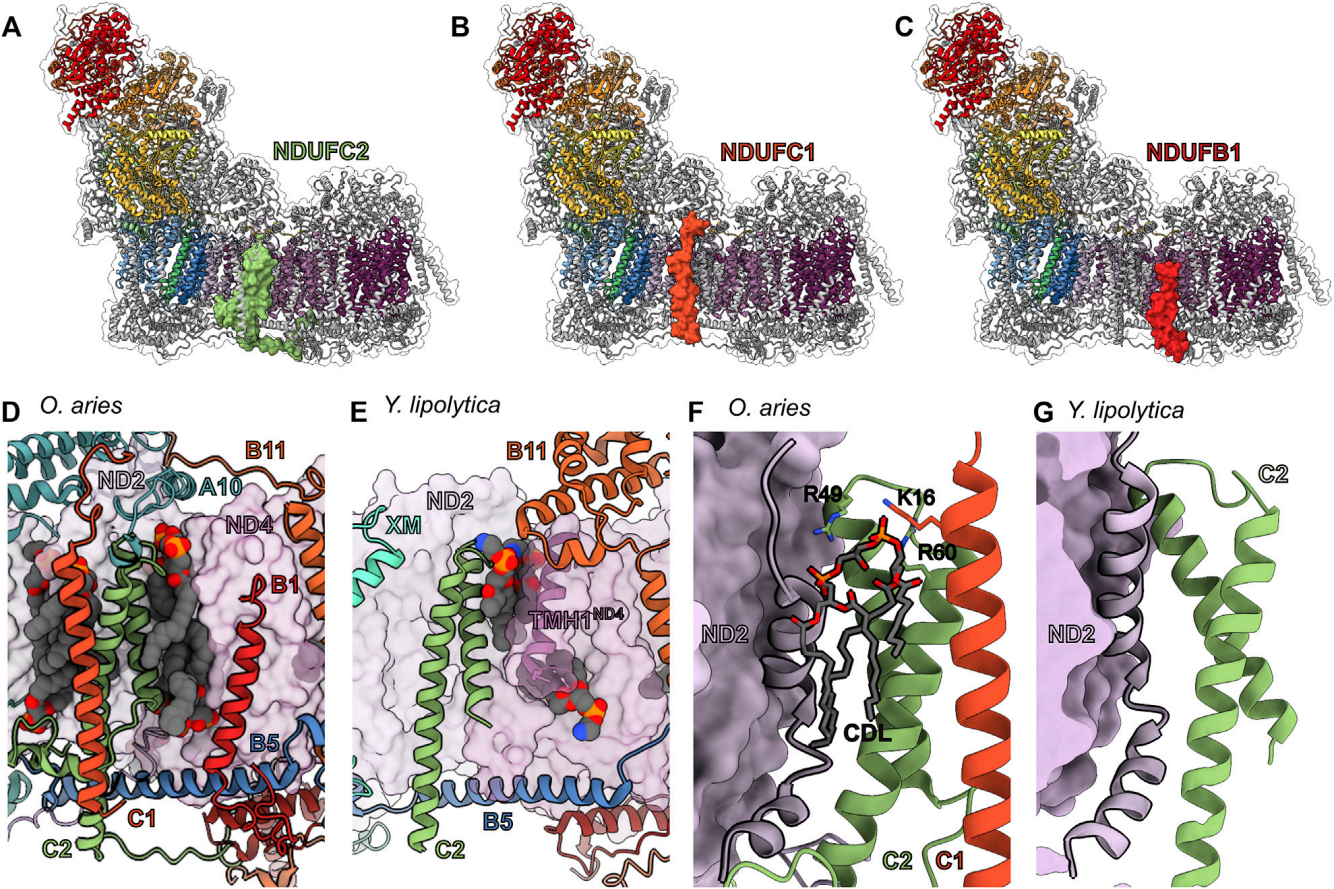
TM accessory subunits of the ND2 and ND4 interface. The structure of mammalian CI (PDB: 6ZKC throughout) in cartoon and the surface of **(A)** NDUFC2 (green), **(B)** NDUFC1 (deep orange) and **(C)** NDUFB1 (red) shown, the core subunits colored as in Figure 1A and the accessory subunits grey. **(D)** Lipid binding pocket in the mammalian ND2 (thistle purple)/ND4 (pearly purple) interface with the accessory subunits in cartoon and lipids in spheres. **(E)** Lipid binding in the *Y. lipolytica* (PDB: 6YJ4) ND2/ND4 interface with the accessory subunits in cartoon and lipids in spheres. **(F,G)** NDUFC1 alters the structure of ND2 through lipid binding. **(F)** NDUFC2 and NDUFC1 form a cardiolipin binding pocket with the final TMH^ND2^ in *O. aries*, TMH11^ND2^ (PDB: 6ZKC). Positive residues that interact with the cardiolipin, as well as the cardiolipin, are represented as sticks. **(G)** The cardiolipin pocket is not present in *Y. lipolytica* which lacks NDUFC1 (PDB: 6YJ4). NDUFC2: green, NDUFC1: deep orange, ND2: thistle purple, ND4: pearly purple, NDUFB11: carrot orange, NDUFB5: cyan azure, NUXM: light cyan.

The interface of core antiporter-like subunits ND2 and ND4 on the side opposite to the ND5 lateral helix (ND5-HL) is defined by a deep lipid filled pocket bordered by the accessory TM subunits NDUFC2, NDUFC1 and NDUFB1 (Figures 10A–D) and capped by NDUFA10.

*NDUFC2*—Along with NDUFC1, NDUFC2 joins ND2, ND3, ND4L, and ND6 to form the P_P_-b subassembly (Figure 2A) (Sánchez-Caballero et al., 2016). NDUFC2 is composed of a N-terminal coil followed by two TMHs bound to ND2 and a C-terminal coil that reaches to ND4 (Figures 10A,D). In the IMS, the N-terminal coil of NDUFC2 occupies space at the interface of the IMS and IMM that is occupied by the first three ND2 TMH’s in non-metazoans. This coil interacts with ND2, NDUFB5, NDUFS5, NDUFA8, and NDUFC1. In the membrane, TMH1^C2^ interacts with ND2 *via* TMH11^ND2^ and TMH9^ND2^, as well as stabilizes several lipid molecules at the interface of ND2 and ND4 (Figure 10D). In the matrix, the TMH1-2^C2^ loop interacts with ND2 and NDUFA10 as well as binds several lipids. TMH2^C2^ interacts with NDUFC1 and NDUFA8. In the IMS, the C-terminal coil of NDUFC2 interacts with NDUFB5, NDUFB10, NDUFB11, and ND4. In HEK293T cells, NDUFC2^KO^ blocks CI assembly and results in a decrease in the abundance of subunits associated with the P_P_-b subassembly and the N-module (Stroud et al., 2016). A heterozygous NDUFC2^KO^ in rats causes an increase in ROS and mitochondrial dysfunction (Raffa et al., 2017). A similar phenotype was observed in another rat model where the reduction of *Ndufc2* expression induced CI dysfunction and promoted stroke-like episodes (Rubattu et al., 2016). These data demonstrate that NDUFC2 is vital for proper CI assembly and activity.

Across species there are notable differences in the structure of NDUFC2’s N- and C-terminal coils. The N- and C-termini in plants and yeast are truncated relative to mammals without any extended coil structures (Figures 10D,E) (Parey et al., 2019; Maldonado et al., 2020). Although the coil structures vary between mammalian species, the presence of the extended termini is conserved in mammals. Notably, the extended C-terminus of NDUFC2 in mammals bridges the P_P_- and P_D_-modules, stabilizing the membrane arm of the complex. A universal feature of NDUFC2 seen in plants, yeast and mammals is the stabilization of lipids both on the surface of ND2 and in a deep pocket at the interface between ND2 and ND4 (Figures 10D,E). This suggests that the main function of NDUFC2 is the stabilization of structured lipids at the interface of ND2 and ND4 and that NDUFC2’s role in bridging between the P_P_-b and P_D_-a subassemblies *via* the extended C-terminal coil evolved later in metazoans.

*NDUFC1*—Similar to NDUFC2, NDUFC1 is part of the P_P_-b subassembly (Figure 2A) (Sánchez-Caballero et al., 2016). NDUFC1 consists of a single TMH that is bound to CI mainly via interaction with NDUFC2 and NDUFA10 (Figures 10B,D). NDUFC1 is one of the two accessory subunits that does not make direct contact with any of the core subunits (NDUFAB1-α is the other). In the mitochondrial matrix, the N-terminal coil of NDUFC1 binds NDUFA10 (Figure 10D). In the membrane NDUFC1 interacts with NDUFC2 and, along with NDUFC2 and ND2, NDUFC1 forms a cardiolipin binding pocket (Figure 10F). In HEK293T cells, NDUFC1^KO^ blocks CI assembly and results in the buildup of an intermediate lacking the N-module (Stroud et al., 2016). Similar to NDUFC2, NDUFC1^KO^ results in a decrease in the abundance of P_P_-b subassembly and N-module subunits (Stroud et al., 2016). This suggests that both NDUFC2 and NDUFC1 are required for the formation or stability of the P_P_-b subassembly.

Subunits analogous to NDUFC1 are absent in *Y. lipolytica* and plant CI (Wirth et al., 2016; Parey et al., 2019; Grba and Hirst, 2020; Maldonado et al., 2020; Klusch et al., 2021), making it, along with NDUFA10, a metazoan specific subunit. The other major metazoan specific difference in this region is the deletion of the first three helices of ND2 and the loss of subunit NDUX1 (NUXM in *Y. lipolytica*). A less prominent difference between the mammalian and yeast/plant structures in this region is that the final TMH of ND2 in mammals (TMH11^ND2^) is shorter on its N-terminal matrix side by two turns compared to that of *Y. lipolytica* and plants (TMH14^ND2^) (Figures 10F,G). This results in the first turn of the helix in mammals being within the membrane with the positive helix dipole capped by the phosphate of the cardiolipin which is held to the complex by NDUFC1 and NDUFC2 (Figure 10F). The position of this cardiolipin conflicts with the additional helical turns of TMH14^ND2^ in *Y. lipolytica* and plants (Figures 10F,G). Importantly, the TMH10-11^ND2^ loop forms a major interaction interface with NDUFA10 and unwinding of TMH11^ND2^ may influence the position of this loop and hence interaction with NDUFA10. Thus, NDUFC1 may work to recruit a cardiolipin molecule that impacts the structure of ND2 which in turn influences the binding of NDUFA10. In this way, although it does not interact directly with any core subunit, NDUFC1 would influence the assembly of the entire complex.

*NDUFB1*—Along with ND4, NDUFB5, NDUFB6, and NDUFB10, NDUFB1 forms part of the P_D_-a subassembly (Figure 2A) (Sánchez-Caballero et al., 2016). NDUFB1 is composed of a single TMH with a C-terminal loop extending into the IMS (Figures 10C,D). The NDUFB1 TMH contacts ND4 and binds two lipid molecules (Figure 10D). The C-terminal loop of NDUFB1 contacts NDUFB5 and NDUFB10 (Figure 10D). In HEK293T cells, NDUFB1^KO^ blocks CI assembly and decreases the abundance of subunits associated with the P_P_-b, P_D_-a and N subassemblies, with only minor impact on subunits associated with the P_P_-a/Q and P_P_-b subassemblies (Stroud et al., 2016). This indicates that NDUFB1 is needed for the stabilization of the P_P_-b/P_D_-a subassembly and thus NDUFB1^KO^ blocks formation of the full Q/P intermediate required for the addition of the N-module in mammals (Guerrero-Castillo et al., 2017a).

It is important to note that a subunit called MNLL, which is a synonym for NDUFB1, was assigned in the recent full length structures of plant and algal CI (Soufari et al., 2020; Klusch et al., 2021). However, this subunit is not a NDUFB1 (MNLL) homolog, nor does it bind in the equivalent position of NDUFB1. Instead, this plant subunit is a structural homolog of the *Y. lipolytica* subunit NUXM, which does not have a homolog in mammals. For this reason, Maldonado et al. (2020) coined this subunit NDUX1, an important distinction, as NDUFB1 appears to be metazoan specific not having homologs in either fungi or plants.

The structures of mammalian and *Y. lipolytica* CI indicate that NDUFB1’s main function may be to stabilize structural lipids at the interface of ND2 and ND4 (Figure 10D). An evolutionary pressure for trapping lipids at this interface becomes apparent in the *Y. lipolytica* structure, which lacks a NDUFB1 homolog, but instead the first TMH of ND4 is pulled away from the rest of subunit occupying an equivalent position to that of NDUFB1 in mammals (Figure 10E). The repositioning of TMH1^ND4^ in *Y. lipolytica* is likely aided through interaction with the matrix domain of NDUFB11 (NESM) and traps several lipids at the interface of ND2 and ND4 (Figure 10E). The evolution of these two distinct strategies for trapping lipids at this interface indicate the importance of these lipids for CI.

### Transmembrane Subunits at the Interface of ND4 and ND5: NDUFB11, NDUFB5, and NDUFB6

**FIGURE 11:**
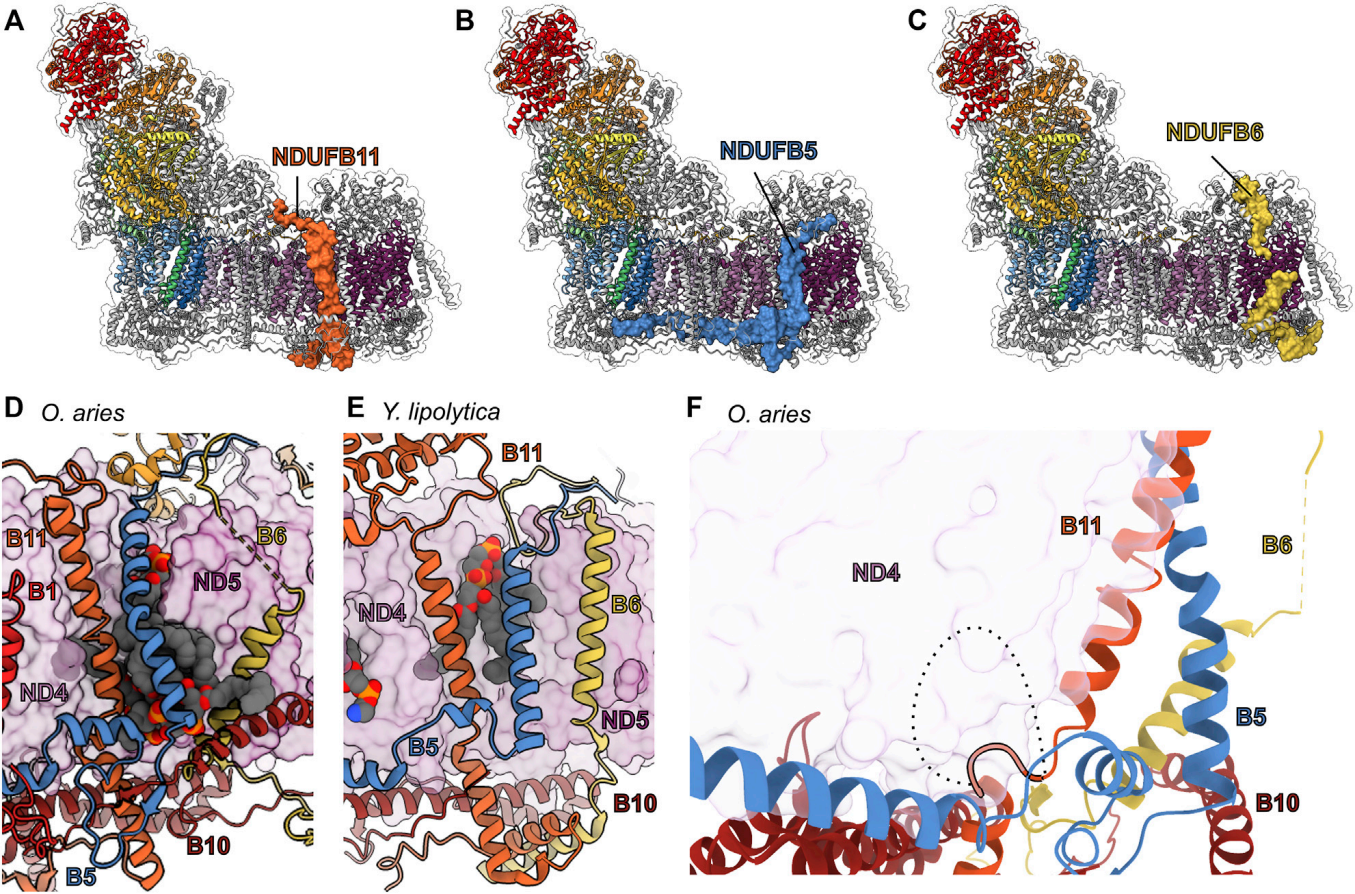
TM accessory subunits of the ND4 and ND5 interface. The structure of mammalian CI (PDB: 6ZKC throughout) in cartoon with the surface of **(A)** NDUFB11 (carrot orange), **(B)** NDUFB5 (cyan azure), and **(C)** NDUFB6 (old gold), the core subunits colored as in Figure 1A and the accessory subunits grey. **(D)** Lipid binding pocket in the mammalian ND4 (pearly purple)/ND5 (boysenberry purple) interface with the accessory subunits in cartoon and lipids in spheres. **(E)** Lipid binding in the *Y. lipoyltica* (PDB: 6YJ4) ND4/ND5 interface with the accessory subunits in cartoon and lipids in spheres. **(F)** The NDUFB11-S (short isoform) is shown in carrot orange cartoon. The additional 10 amino acids of the NDUFB11-L (long isoform, represented as dashed line) clash with core subunit ND4 but may be accommodated in the P_D_-a′ assembly with accessory subunits NDUFB6, NDUFB10 (vivid auburn) and NDUFB11 shown in cartoon. NDUFB11: carrot orange, NDUFB5: cyan azure, NDUFB6: old gold, ND4: pearly purple, ND5: boysenberry purple, NDUFB10: vivid auburn.

Like the interface of ND2 and ND4 discussed above, the interface of the antiporter-like subunits ND4 and ND5 on the side opposite the ND5-HL is also defined by a deep lipid filled pocket. This pocket is bordered by the accessory TM subunits NDUFB11, NDUFB5 and NDUFB6 (Figures 11A–C).

*NDUFB11*—Dynamic complexome profiling of CI assembly indicates NDUFB11 preassembles with subunits NDUFB5, NDUFB6, and NDUFB10 forming what we coin the P_D_-a′ subassembly, before joining ND4 and NDUFB1 to form the P_D_-a subassembly (Figure 2A) (Guerrero-Castillo et al., 2017a). NDUFB11 is a single TMH subunit with a long N-terminal coil in the matrix and a C-terminal α-helix and coil in the IMS (Figures 11A,D). In the matrix NDUFB11’s N-terminal 50 amino acids are disordered. The resolved residues of the N-terminal coil bind across the matrix side of the membrane arm interacting with NDUFA10, NDUFS2, NDUFB4 and NDUFB9. The NDUFB11 TMH predominantly binds ND4 directly, but also on the IMS side of the membrane pins a lipid molecule to the surface of ND4 (Figure 11D). In both mammals and *Y. lipolytica*, NDUFB11 helps to form the side of the lipid filled cavity between ND4 and ND5 (Figures 11D,E). In the IMS NDUFB11 interacts with NDUFB5, ND4, NDUFB10, ND5 and NDUFC2.

In HEK293T cells, NDUFB11^KO^ disrupts CI assembly with a decrease in the abundance of subunits associated with the P_P_-b, P_D_-a, P_D_-b and N subassemblies (Stroud et al., 2016). Mutations in the *Ndufb11* gene, including an in frame deletion of F93^B11^ located in the NDUFB11 TMH that would impact its interaction with ND4, compromise CI stability and have been associated with various diseases such as congenital sideroblastic anemia, microphthalmia with linear skin defects, and lactic acidosis (Van Rahden et al., 2015; Lichtenstein et al., 2016; Torraco et al., 2017). Thus, NDUFB11 is important for CI assembly and activity. Additionally, NDUFB11 is one of the few supernumerary subunits to have isoforms produced by alternative splicing (Panelli et al., 2013). The short 153 amino acid isoform is the structurally resolved CI subunit and is expressed at higher levels than its longer 163 amino acid isoform (Panelli et al., 2013). The isoforms utilize two different 5′ splice sites and the longer 163 amino acid isoform includes an additional 30 nucleotides in the second exon of *Ndufb11* (Panelli et al., 2013) thereby producing a NDUFB11 isoform with an additional 10 amino acid residues inserted into the loop between the TMH and the IMS α-helix (α1^B11^) (Figure 11F). In the fully assembled complex, the TMH-α1^B11^ loop packs closely against ND4 and major conformational changes would be needed to accommodate the additional residues of the splice variant (Figure 11F). Nonetheless, the location of the insertion and the fact that it is predicted to be an unstructured coil may not prevent the formation of the P_D_-a′ subassembly between NDUFB5, NDUFB6, NDUFB10 and NDUFB11 (Figure 11F). In this way, the long NDUFB11 isoform may regulate CI assembly by sequestering the P_D_-a′ interaction partners while blocking association of ND4. Interestingly, treatment of human SH-SY5Y neuroblastoma cells with the specific CI inhibitor rotenone enhances the expression of the long isoform while also triggering apoptosis suggesting a regulatory response to mitochondrial stress or cell death (Panelli et al., 2013). More work is needed to fully understand the role of the NDUFB11 isoforms.

*NDUFB5*—In the fully assembled complex, NDUFB5 spans the membrane arm of the complex with its TMH situated between ND4 and ND5 and two α-helices in the IMS parallel to the membrane (Figure 11B). In the matrix, the N-terminal coil of NDUFB5 contacts NDUFB3, NDUFAB1-β, NDUFB6, NDUFB9, and ND5. In the membrane, NDUFB5 contacts ND4, NDUFB11 and several well resolved lipid molecules. The NDUFB5 TMH pins NDUFB11 and the lipid molecules onto the surfaces of ND4 and ND5. In the IMS, NDUFB5 wraps around NDUFB10 with a coil of NDUFB10 threading through an eyelet formed by a coil of NDUFB5 (Figures 11D,F). This eyelet is not present in the *Y. lipolytica* structure and both NDUFB10 and the NDUFB5 loop (TMH-α1^B5^ loop) are shorter in yeast CI (Figures 11D,E). This interaction is directly followed by a short α-helix (α1^B5^) which binds overtop NDUFB11 and contacts ND4, then a long α-helix (α2^B5^) which contacts NDUFB1, ND4, NDUFB10, NDUFA8, NDUFC2, NDUFA11, and ND2. Finally, the C-terminal coil of NDUFB5 contacts NDUFC2, ND2, NDUFS5, NDUFA8, NDUFA3, and NDUFA13 (Figure 8H). Given this extensive network of interactions that span the membrane arm, it is not surprising that in HEK293T cells NDUFB5^KO^ blocks CI assembly with a larger impact in the abundance of subunits in the P_P_-b and P_D_-a subassemblies (Stroud et al., 2016) compared to the Q or P_D_-b subassemblies. This indicates that NDUFB5 is essential for establishing the P_D_-a subassembly and formation of the P_P_-b/P_D_-a subassembly (Stroud et al., 2016). NDUFB5’s long α2^B5^ which binds along the IMS side of the membrane arm likely plays the major role in the connection between the P_D_-a and P_P_-b modules (Figure 11B).

In both mammals and *Y. lipolytica* the NDUFB5 (NUUM in *Y. lipolytica*) TMHs cap a lipid filled cavity trapping several lipids at the interface between ND4 and ND5. This indicates an important role for NDUFB5 in trapping lipids at the interface of ND4 and ND5 in addition to its role in assembly. Interestingly, in plants the accessory subunit P2 is structurally analogous to the long IMS helix of NDUFB5 (α2^B5^) but lacks the TMH (Soufari et al., 2020; Klusch et al., 2021). This indicates that the roles of NDUFB5 in assembly and lipid binding may be separable, with plants using this subunit only to aid complex assembly and stability and opisthokonts using it additionally for lipid sequestration.

*NDUFB6*—Despite its low sequence conservation, NDUFB6 is structurally conserved across eukaryotes. NDUFB6 is a single TMH subunit that has an N-terminal matrix domain containing an α-helix (α1^B6^) which forms part of the matrix P_D_-bulge (Figure 1A), a highly tilted TMH which is partially disordered in mammals, indicating flexibility, and an IMS C-terminal coil (Figure 11C). Although in mature CI NDUFB6 mostly interacts with the subunits of the P_D_-b subassembly, during assembly it joins the P_D_-a′ subassembly and then the P_D_-a subassembly (Figure 2A) (Sánchez-Caballero et al., 2016). In the fully assembled complex, NDUFB6 interacts with NDUFAB1-β, NDUFB9 and NDUFB5 as part of the matrix P_D_-bulge (Figure 6C). The matrix side of the NDUFB6 TMH is disordered in mammals with only the IMS half of the TMH having clearly defined density, indicating a flexibility in the matrix half of the TMH not seen for any other TM accessory subunit. The IMS half of NDUFB6’s TMH interacts with ND5 and NDUFB10. Together with NDUFB5 and NDUFB11, NDUFB6’s TMH^B6^ forms the boundary of a lipid filled cavity at the interface between ND4 and ND5 (Figure 11D). In the IMS the C-terminal coil of NDUFB6 interacts with ND5, NDUFB10, and NDUFB7.

In HEK293T cells, NDUFB6^KO^ prevents full assembly of the complex and reduces the abundance of subunits of the N-, Q-, and P_D_-b modules (Stroud et al., 2016) and NDUFB6 depletion in HEK293T Flp-In cells displayed an 80% decrease in CI activity (Loublier et al., 2011) indicating that NDUFB6 is necessary for CI assembly and hence activity. Moreover, NDUFB6 expression may play a regulatory role in CI activity. Patients with type 2 diabetes mellitus experience a decrease in NDUFB6 expression in muscle cells (Ling et al., 2007). DNA methylation in the NDUFB6 promoter present in elderly patients was observed to reduce NDUFB6 expression, suggesting an epigenetic-basis of NDUFB6 regulation (Ling et al., 2007).

Given that NDUFB6 forms part of the P_D_-a subassembly but that it interacts mostly with P_D_-b subassembly subunits in the final CI structure and NDUFB6^KO^ results in decreased levels of subunits associated with the P_D_-b subassembly, it is clear that a major role of NDUFB6 is to bridge the P_D_-a and P_D_-b subassemblies during CI biogenesis in mammals. As NDUFB6 does not directly interact with ND4, this bridging interaction would be facilitated through the interactions with the P_D_-bulge in the matrix and the P_D_-a′ subassembly subunits, specifically NDUFB10, in the IMS.

### The Sole of CI—Intermembrane Space CX_9_C Motif Subunits

**FIGURE 12:**
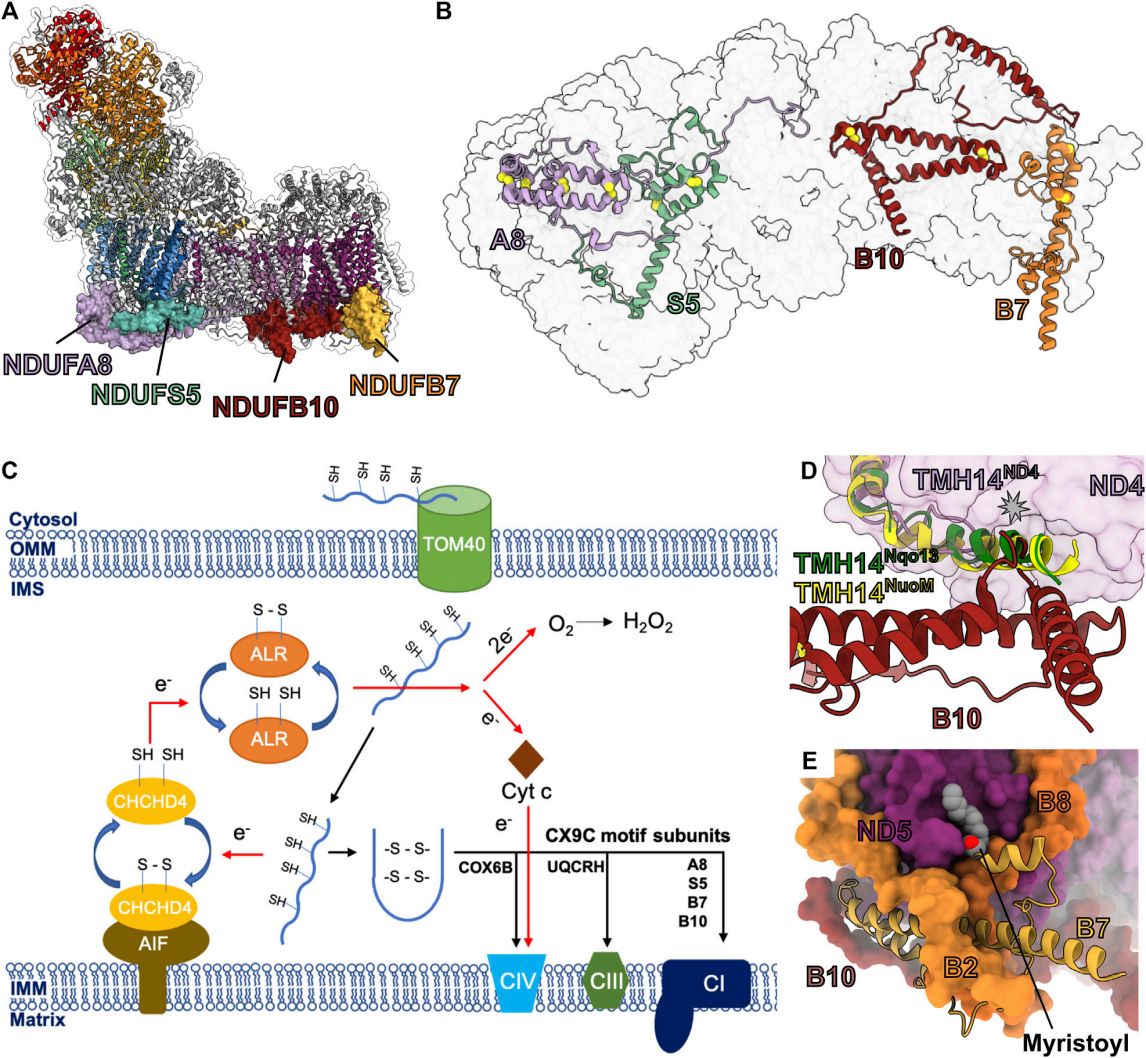
The CX_9_C motif containing accessory subunits. **(A)** The structure of mammalian CI (PDB: 6ZKC throughout unless stated otherwise) in cartoon with the surface of NDUFA8 (lilac purple), NDUFS5 (cadet blue), NDUFB10 (vivid auburn) and NDUFB7 (gold) shown, the core subunits colored as in Figure 1A and the other accessory subunits grey. **(B)** IMS view of the CI sole showing the CX_9_C motif containing subunits. CI is shown as a transparent surface. The CX_9_C subunits are shown as cartoons colored as in Figure 1 with the disulfide bonding residues shown as spheres. **(C)** Schematic diagram showing the import of CX_9_C motif subunits into the IMS and assembly onto CI. CX_9_C motif subunits are imported from the cytosol into the IMS via outer membrane protein TOM40. CX_9_C subunits are oxidized by CHCHD4 protein bound to the AIF protein in its NADH bound state. The folded CX_9_C subunits are assembled on their destined ETC complex. The electrons are transferred from CHCHD4 protein to CIV *via* the ALR protein and cyt *c* or are transferred from ALR protein to molecular O_2_. **(D)** NDUFB10 interacts with the surface of ND4 (pearly purple) and compensates for the loss of the extended helical region of TMH14^ND4^ present in bacteria. ND4 (NuoM) of *E. coli* CI (PDB: 7NZ1) and ND4 (Nqo13) of *T. thermophilus* (PDB: 4HEA) are superposed onto ND4 of mammalian (*O. aries*) CI structure. NDUFB10 and TMH14^ND4^ are shown as cartoons. **(E)** NDUFB7 is myristoylated. Mammalian NDUFB7 (PDB: 6ZKO) of CI is shown as cartoon and other subunits are shown as colored surface. The myristoyl group is shown as spheres. NDUFA8: lilac purple, NDUFS5: cadet blue, NDUFB10: vivid auburn, NDUFB7: gold, ND4: pearly purple, ND5: boysenberry purple.

NDUFA8, NDUFS5, NDUFB10, and NDUFB7 are members of the coiled-coil-helix-coiled-coil-helix domain-containing family that carry CX_9_C motifs, and all reside on the IMS surface of the CI membrane arm (Figures 12A,B). NDUFA8 is a subunit of the P_P_-a subassembly added to ND1 along with NDUFA3 and NDUFA13 (Figure 2A). NDUFS5 is a subunit of the P_P_-b/P_D_-a subassembly and assembles along with NDUFA10 and NDUFB4 upon connection of P_D_-b and P_D_-a (Figure 2A). NDUFB10 is part of the P_D_-a′ subassembly with NDUFB11, NDUFB5 and NDUFB6 and goes on to form the P_D_-a subassembly along with the addition of ND4 and NDUFB1 (Figure 2A). NDUFB7 is a subunit of the P_D_-b subassembly, it assembles with ND5, NDUFB2, NDUFB3, NDUFB7, NDUFB8, NDUFB9, and NDUFAB1-β (Figure 2A) (Guerrero-Castillo et al., 2017a). Thus, during assembly each of the membrane arm subassemblies has an associated CX_9_C motif subunit: P_P_-a has NDUFA8, P_P_-b has NDUFS5, P_D_-a has NDUFB10, and P_D_-b has NDUFB7 (Figure 2A).

The CX_9_C motifs of these subunits form disulfide bonds that stabilize the helix-turn-helix structure of the subunits (Figure 12B) (Ugalde et al., 2004; Longen et al., 2009; Szklarczyk et al., 2011). These subunits are imported into the IMS in an unfolded reduced form and subsequently folded and oxidized via the disulfide relay-dependent Mitochondrial Import and Assembly (MIA) pathway (Figure 12C) (Mesecke et al., 2005; Fischer et al., 2013; Modjtahedi et al., 2016; Dickson-Murray et al., 2021). The electrons thus released during the oxidation are fed to CIV of the ETC via cytochrome *c* (cyt *c*) or released as reactive oxygen species (ROS) (Figure 12C) (Allen et al., 2005; Bihlmaier et al., 2007). The MIA pathway is regulated by the redox environment of the cell and interacts with several antioxidant systems (Nakao et al., 2015; Kritsiligkou et al., 2017), as well as small molecule regulators (Dickson-Murray et al., 2021).

*NDUFA8*—In the fully assembled complex NDUFA8 binds overtop NDUFA13 forming the base of CI’s heel (Figure 8D and Figure 12A). NDUFA8 has an N-terminal coil, followed by six α-helices folded into an L-shape containing two disulfide cross-linked helix-turn-helix motifs (Figure 12B) and a long C-terminal coil. The N-terminal region of NDUFA8 interacts with NDUFA13, NDUFS5 and NDUFA1. The first NDUFA8 disulfide cross-linked helix-turn-helix motif (α3-α4^A8^) interacts with NDUFA13, NDUFB5 and NDUFA3. The second NDUFA8 disulfide cross-linked helix-turn-helix motif (α5-α6^A8^) interacts with NDUFA1, ND1 and NDUFA13. The C-terminal coil of NDUFA8 reaches halfway down the membrane arm interacting with NDUFA3, NDUFA13, NDUFS5, NDUFB5, NDUFC2, ND2 and ND4. The C-terminus of NDUFA8 also interacts with lipid molecules bound at the interface of ND2 and ND4.

*NDUFS5*—Spanning the IMS side of the P_p_-b module, NDUFS5 interacts with core subunits ND6, ND4L and ND2 (Figures 12A,B). In mammals NDUFS5’s N-terminal coil occupies space at the interface of the IMS and IMM that is occupied by the first three ND2 TMHs in other species. This is followed by the disulfide cross-linked helix-turn-helix motif (α1-α2^S5^), a α-helix (α3^S5^) and a C-terminal coil (Figure 12B). The N-terminal coil of NDUFS5 interacts with ND2, NDUFC2, NDUFB5, ND4L, ND6 and NDUFA3. The disulfide cross-linked NDUFS5 helix-turn-helix motif interacts with NDUFA8, NDUFB5, ND2 and ND4L. NDUFS5’s α3^S5^ interacts with ND4L, ND6 and NDUFA13. The C-terminal coil of NDUFS5 interacts with NDUFA13, NDUFA1, and NDUFA8.

*NDUFB10*—During CI assembly NDUFB10 associates with NDUFB5, NDUFB6 and NDUFB11 to form the P_D_-a′ subassembly before interacting with any core subunits (Figure 2A). In the fully assembled complex NDUFB10 zigzags between ND5 and ND4 interacting with both core subunits and several accessory subunits (Figures 12A,B). NDUFB10’s N-terminal coil and helix interact with NDUFB11, NDUFB6, NDUFB7, ND5 and NDUFB5. The long α1-2^B10^ loop passes through an eyelet formed by NDUFB5 and interacts with NDUFB11, NDUFB1, and NDUFC2. The disulfide cross-linked NDUFB10 helix-turn-helix motif interacts with ND4, NDUFB11, ND5, NDUFB6, NDUFB7, NDUFB8 and NDUFB4. The C-terminal region of NDUFB10 interacts with ND4, NDUFC2 and NDUFB11. NDUFB10 occupies a binding site on the bottom of ND4 that in both *T. thermophilus* and *E. coli* CI structures is occupied by extensions of ND4 TMH^14^, suggesting that interactions in the pocket formed by the TMH8-9^ND4^, TMH10-11^ND4^, and TMH11-12^ND4^ loops may be important for stability of the complex (Figure 12D).

*NDUFB7*—Located under the CI toe, NDUFB7 has a long N-terminal coil with two short α-helices (α1^B7^ and α2^B7^) followed by the disulfide cross-linked helix-turn-helix motif (α3-α4^B7^) (Figures 12A,B). The N-terminal glycine of NDUFB7 is myristoylated anchoring it to IMS leaflet of the membrane (Figure 12E) (Carroll et al., 2005). NDUFB7 is the only CI subunit that is known to be lipid modified. The myristoyl group is bound in a groove on ND5 formed by TMH12^ND5^, TMH13^ND5^ and TMH15^ND5^ (Figure 12E) (Kampjut and Sazanov, 2020). The N-terminal coil of NDUFB7 interacts with ND5, NDUFB8, NDUFB2, NDUFB6 and NDUFB10. The NDUFB7 disulfide cross-linked helix-turn-helix motif interacts with ND5, NDUFB10, NDUFB6 and NDUFB2.

*Roles of NDUFA8, NDUFS5, NDUFB10 and NDUFB7 in CI assembly—*In HEK293T cells, NDUFA8^KO^, NDUFS5^KO^, NDUFB10^KO^ or NDUFB7^KO^ results CI assembly defects and reductions in subunits associated with the P_P_-a, P_P_-b, P_D_-a and P_D_-b subassemblies respectively as well as N-module subunits (Stroud et al., 2016). Accordingly, CI activity and basal mitochondrial respiration are reduced drastically in all four KOs (Stroud et al., 2016). NDUFS5 (Nuo-11.5) and NDUFA8 (Nuo-20.8) deletion in *N. crassa* prevents full assembly of CI and results in the accumulation of membrane arm intermediates (Vieira Da Silva et al., 1996; Marques et al., 2007). A missense mutation in NDUFB10 (C107S^B10^) was discovered in patients with CI deficiency (Friederich et al., 2017). The substitution of the highly conserved C107^B10^, which is involved in disulfide crosslinking, blocks import of the mutated protein into the IMS due to its failure to act as a CHCHD4 substrate (Figure 12C) (Friederich et al., 2017). These results indicate that NDUFA8, NDUFS5, NDUFB10 and NDUFB7 are each essential for CI assembly and stability.

The dependence on CX_9_C motif containing subunits ties CI assembly to the functioning of the MIA pathway which in turn depends upon pre-existence of a functional ETC, namely sufficient CIV to act as an electron sink in the disulfide relay (Figure 12C). Additionally, the MIA pathway is highly regulated through redox signaling (Dickson-Murray et al., 2021). Hence, the evolution of essential CX_9_C motif containing subunits has multiple applications: 1) they enhance CI stability through rigid disulfide linked structures, 2) they ensure that CI assembly only occurs in healthy respiring mitochondria and 3) they allow for additional regulation of CI assembly by the overall redox status of the cell. It is important to note that both CIII and CIV also have CX_9_C motif containing subunits (UQCRH and COX6B respectively) indicating that this is a general strategy used for the entire ETC (Figure 12C).

### Transmembrane Subunits at the Tip of the Toe: NDUFB2 and NDUFB3

**FIGURE 13:**
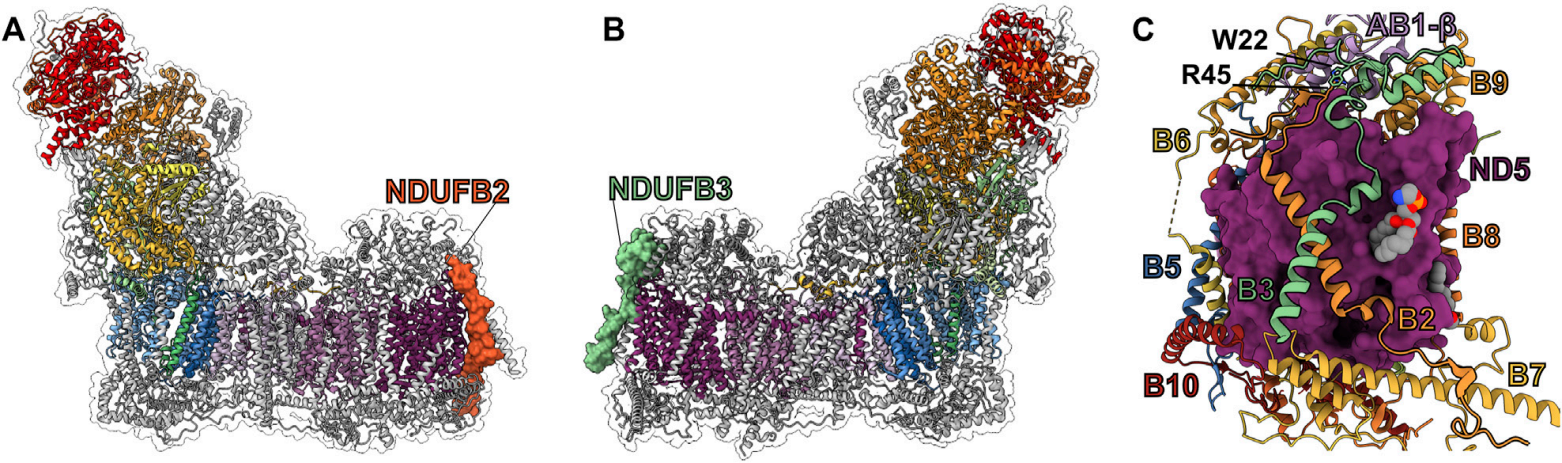
TM accessory subunits at the CI toe. The structure of mammalian CI (PDB: 6ZKC) in cartoon with the surface of **(A)** NDUFB2 (yellow orange) and **(B)** NDUFB3 (iguana green) shown, the core subunits colored as in Figure 1A and the other accessory subunits grey. **(C)** Cartoon of accessory subunits on surface of ND5 (boysenberry purple). Key residue mentioned in the text are shown. Lipid and NDUFB7 myristoyl group are shown as spheres. NDUFB2: yellow orange, NDUFB3: iguana green, ND5: boysenberry purple.

NDUFB2 and NDUFB3 are single TMH subunits bound to the toe of the CI membrane arm (Figures 13A,B). During assembly they are incorporated together as part of the P_D_-b subassembly with core subunit ND5 and accessory subunits NDUFAB1-β, NDUFB7, NDUFB8, and NDUFB9 (Figure 2A) (Stroud et al., 2016; Guerrero-Castillo et al., 2017a).

*NDUFB2*—In the matrix, NDUFB2’s N-terminal coil interacts with NDUFAB1-β, NDUFB3 and ND5 (Figure 13C). On the matrix side of the membrane, NDUFB2’s TMH binds between TMH12^ND5^ and TMH14^ND5^ interacting with the extended loop of the broken THM12^ND5^. In the IMS, NDUFB2’s C-terminal coil interacts with ND5 and NDUFB7 (Figure 13C).

*NDUFB3*—The N-terminal matrix domain of NDUFB3 interacts with NDUFAB1-β, NDUFB9, NDUFB2, and ND5 (Figure 13C). In the membrane, the N-terminus of NDUFB3’s TMH binds in a pocket on ND5 formed by TMH12^ND5^, TMH13^ND5^ and TMH15^ND5^ then as it encounters NDUFB2 it kinks sharply and extends outward from the tip of the membrane arm with no visible structure extending into the IMS (Figures 13B,C). Thus, NDUFB3 pins NDUFB2 to the surface of ND5. The N-terminal tilted region of NDUFB3’s TMH creates a lipid binding site on the surface of ND5 in which the lipid is pulled down relative to the plane of the membrane indicating thinning of the membrane in this region (Figure 13C).

*Roles of NDUFB2 and NDUFB3 in CI assembly*—In HEK293T cells, NDUFB2^KO^ or NDUFB3^KO^ results in a severe CI assembly defect with a decrease in the abundance of P_D_-b and N subassembly subunits (Stroud et al., 2016). The NDUFB2 gene contains a highly conserved CHOP element in its promoter (Aldridge et al., 2007). Transcription of CHOP element containing genes is upregulated in response to the accumulation of unfolded proteins and induce the mitochondrial unfolded protein response (Aldridge et al., 2007). A point mutation in NDUFB3 (W22R^B3^) is associated with CI deficiency (Calvo et al., 2012; Haack et al., 2012) as well as with a distinctive facial appearance and short stature (Alston et al., 2016). W22 is located on the N-terminal matrix loop of NDUFB3, and binds overtop of NDUFB2 R45^B2^, the W22R^B3^ mutation would introduce unfavorable electrostatics likely disrupting the interactions in this region (Figure 13C). Therefore, the W22R^B3^ mutation reveals the importance of the NDUFB2 and NDUFB3 interactions in the mitochondrial matrix for overall CI activity.

NDUFB3 is also involved with supercomplex formation as it contacts CIV (via COX8B) in the tight conformation of the respirasome (Letts et al., 2016b). Deformation of the lipid bilayer by NDUFB2 at this interface may promote interaction with CIV. Altogether, due to their many interactions with accessory subunits on both sides of the membrane, NDUFB2 and NDUFB3 are required for the assembly of the P_D_-b subassembly and are hence essential to CI biogenesis.

### Transmembrane Subunits of the Lateral Helix: NDUFB8, NDUFB4 and NDUFA11

**FIGURE 14:**
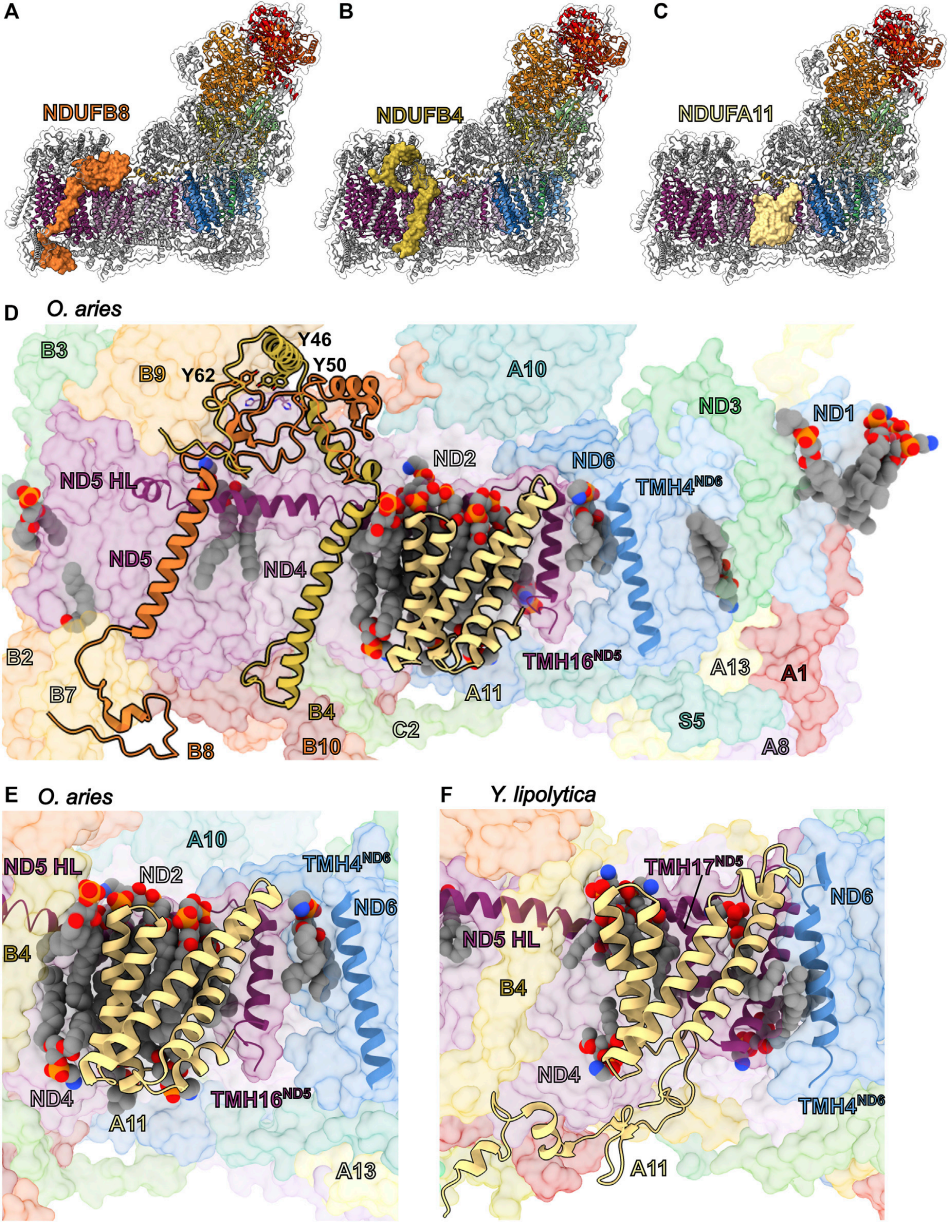
TM accessory subunits of the ND5 lateral helix (ND5 HL). The structure of mammalian CI (PDB: 6ZKC throughout unless stated otherwise) in cartoon with the surface of **(A)** NDUFB8 (vivid tangelo), **(B)** NDUFB4 (old gold) and **(C)** NDUFA11 (wheat) shown, the core subunits colored as in Figure 1A and the other accessory subunits grey. **(D)** Mammalian NDUFB8, NDUFB4 and NDUFA11 subunits are shown as cartoons on the surface of CI. Residues that are discussed in the text are shown as sticks. ND5 HL, TMH16^ND5^ and TMH4^ND6^ are shown as cartoon. Lipids are shown as spheres. **(E)** Zoom in on mammalian NDUFA11 shown as cartoon. ND5 HL, TMH16^ND5^ and TMH4^ND6^ are shown as cartoon. Lipids are shown as spheres. **(F)** *Y. lipolytica* NDUFA11 (NUJM; PDB: 6RFR) shown as cartoon. The ND5 (NU5M) HL (ND5 HL), TMH16^ND5(NU5M)^, TMH17^ND5(NU5M^ and TMH14^ND6(NU6M)^ are shown as cartoons. The lipids are shows as spheres. Subunits are colored as in Figure 1 unless stated otherwise. NDUFB8: vivid tangelo, NDUFB4: old gold, NDUFA11: wheat, ND5: boysenberry purple, ND6: cyan-blue.

The lateral helix of ND5 (ND5 HL) is an amphipathic helix that stretches between TMH15^ND5^ at the tip of the membrane arm and TMH16^ND5^ adjacent to ND2 and ND4L (Figure 1A). Along its length ND5-HL is bound by three accessory transmembrane subunits, NDUFB8, NDUFB4 and NDUFA11 (Figures 14A–C). These subunits all bind overtop of ND5-HL and help anchor it to the complex.

*NDUFB8*—Together with ND5, NDUFB2, NDUFB3, NDUFB7, NDUFB9, and NDUFAB1-β, NDUFB8 forms the P_D_-b subassembly (Figure 2A) (Guerrero-Castillo et al., 2017a). NDUFB8 is a single TMH accessory subunit with an N-terminal domain in the matrix and a short C-terminal coil in the IMS. Despite structural variability in the N- and C-terminal regions, the position of NDUFB8 is conserved in mammals, yeast and plants (Parey et al., 2019; Kampjut and Sazanov, 2020; Klusch et al., 2021). In mammals, the N-terminal domain interacts with NDUFB4, ND4, ND5, NDUFB9 and NDUFAB1-β, as well as with the head group of a lipid that is trapped by the ND5-HL at the ND5/ND4 interface (Figure 14D). In the membrane NDUFB8 interacts solely with ND5. In the IMS the C-terminal coil of NDUFB8 interacts with ND5, NDUFB7, NDUFB10 and NDUFB2.

In HEK293T cells, NDUFB8^KO^ prevents proper assembly of CI and results in a decreased abundance of subunits associated with the N-module (Stroud et al., 2016). NDUFB8 mutations Y62H^B8^, E63D^B8^, P76Q^B8^, C144W^B8^ or deletion of the region M105-V156^B8^ cause CI deficiency with symptoms ranging from fatal infantile lactic acidosis to Leigh-like syndrome (Piekutowska-Abramczuk et al., 2018). In humans, C144^B8^ (not conserved in other *sps*.) is positioned to form a possible inter-subunit disulfide bond with core subunit ND5 residue C279^ND5^. The larger tryptophan sidechain in C144W^B8^ mutation would disrupt the close interaction between NDUFB4 and ND5 in this region. The other mutations are in the matrix domain of the complex and would disrupt the stability of the P_D_-bulge.

*NDUFB4*—During assembly of CI NDUFB4 does not form part of the individual membrane arm subassemblies but has been proposed to join during formation of the P_P_-b/P_D_-a subassembly or the Q/P subassembly (Figure 2A) (Guerrero-Castillo et al., 2017a). In the fully assembled complex the N-terminal matrix domain of NDUFB4 is composed of a coil and two helices (α1^B4^ and α2^B4^) that zigzag over the matrix side of the complex forming part of the P_D_-bulge and interacting with NDUFB9, NDUFB8, ND5, NDUFB11 and ND4 (Figures 14B,D). In the membrane, NDUFB4 interacts with ND5 via ND5-HL, and ND4. NDUFB4 along with NDUFA11 also traps lipids to the surface of the complex (Figure 14D). In the IMS, NDUFB4 interacts with NDUFC2, NDUFB10, ND5 and ND4.

The position of NDUFB4 binding overtop of ND5-HL raises questions about the proposed assembly pathway (Guerrero-Castillo et al., 2017a). If NDUFB4 binds to the P_P_-b/P_D_-a subassembly, unless its interaction with ND4 is very different than what is observed in the structure of the full complex, it would block the binding of the ND5-HL preventing the addition of the P_D_-b subassembly. Thus, the structure indicates that NDUFB4 would need to be added only after the connection of the P_D_-a and P_D_-b subassemblies, which occurs upon formation of the Q/P subassembly (Figure 2A). This also explains why NDUFB4 is not part of any of the individual membrane arm subassemblies but is only added to the complex later. Together with NDUFB9, NDUFB4 forms one of the most extensive contacts with CIII_2_ in the supercomplex, binding to a loop extended from the UQCRC1 subunit of CIII_2_ (Letts et al., 2019).

In HEK293T cells, NDUFB4^KO^ results in incomplete assembly of the complex with a reduction in the levels of subunits in the P_D_-b subassembly and N-module (Stroud et al., 2016). NDUFB4 can be modified by peroxynitrite resulting in 3-nitrotyrosine at Tyr46^B4^, Tyr50^B4^ and Tyr51^B4^ in *B. taurus* (Murray et al., 2003). Tyr46 and Tyr50 are conserved in humans and in *O. aries* on α1^B4^ in the matrix (Figure 14D). In *O. aries* Tyr46 and Tyr50 contact ND4 (*via* His421^ND4^, His422^ND4^ of the TMH13-14^ND4^ loop), NDUFB9 (*via* His168^B9^) and NDUFB8 (*via* Asp65^B8^ and Trp73^B8^). Tyr46 and Tyr50 are hypothesized to contribute to the altered function of CI and perhaps play a role in the onset of Parkinson’s disease (Murray et al., 2003). Overall, this suggests an important role for the NDUFB4 P_D_-bulge interactions in CI assembly and stability and leads to the hypothesis that NDUFB4 acts to cement the interaction between the P_D_-a and P_D_-b subassemblies during CI biogenesis. This is achieved by NDUFB4 “clamping” over ND5-HL, intertwining with the P_D_-bulge in the matrix and binding ND4, ND5 and NDUFB10 in the IMS (Figure 14D).

*NDUFA11*—The four TMH subunit NDUFA11 binds overtop of ND5-HL adjacent to the final TMH16^ND5^ at the interface of ND2 and ND4 (Figure 14C). During CI assembly NDUFA11 joins the complex only after formation of the Q/P-subassembly making it the final membrane arm subunit to join the complex (Figure 2A) (Sánchez-Caballero et al., 2016). NDUFA11 is structurally conserved across eukaryotes and is homologous to Tim17, Tim22, and Tim23, which are involved in protein translocation across the inner membrane (Carroll et al., 2002). NDUFA11’s four TMHs form an “arch” shape with the feet of the arch contacting CI and the central cavity of the arch filled with lipids (Figure 14D). It interacts with core subunits ND2, ND4 and ND5 with most of these interactions mediated *via* lipids trapped between NDUFA11 and the core subunits. The C-terminal coil of NDUFA11 interacts with ND2, ND4 and NDUFB5. In addition to the lipids trapped between NDUFA11 and ND2, ND4 and ND5, NDUFA11 also binds lipids adjacent to NDUFB4 and ND6 TMH4^ND6^ (Figure 14D).

Suppression of NDUFA11 expression in human osteosarcoma 143B cells results in reduced oxygen consumption, a fragmented mitochondrial network, a reduction in intact CI and accumulation of subassemblies associated with assembly factors NDUFAF1-4, ACAD9, ECSIT, FOXRED1 and TMEM126B (Andrews et al., 2013). In HEK293T cells, NDUFA11^KO^ results in incomplete assembly of CI with a decrease in the levels of subunits associated with the N-module (Stroud et al., 2016) indicating that NDUFA11 is needed for the final stages of CI biogenesis.

In *Y. lipolytica* ND5 has two TMHs after ND5-HL (TMH16^ND5^ and TMH17^ND5^) whereas mammalian ND5 on has one (TMH16^ND5^) (Figures 14E,F). The additional TMH17^ND5^ binds within the central cavity of the NDUFA11 arch displacing some of the lipids seen in the mammalian structures (Figures 14E,F). Also, *Y. lipolytica* NDUFA11 has a longer C-terminal coil relative to mammals, that extends along the IMS side of the complex interacting with ND4, NDUFB10, NDUFB4, NDUFB11, and NDUFB6 (Figure 14F). In *Y. lipolytica* and bacterial CI structures (*E. coli* and *T. thermophilus*) TMH4^ND6^ binds at the interface of TMH16^ND5^, TMH1^ND4L^ and TMH6^ND2^, forming additional contacts with NDUFA11 in *Y. lipolytica* (Figure 14F). In mammals, TMH4^ND6^ is displaced from this position binding adjacent to TMH1^ND6^ and not directly interacting with ND2 or NDUFA11 (Figure 14E). These additional NDUFA11 interactions in *Y. lipolytica* are likely why dissolution of CI using the harsh detergent lauryl dimethylamine oxide (LDAO) results in a different pattern of subcomplexes in *Y. lipolytica* vs. mammals (reviewed by Letts and Sazanov 2015) (Sazanov et al., 2000; Angerer et al., 2011; Letts and Sazanov, 2015; Letts et al., 2016a). In *Y. lipolytica* the P_D_-module remains associated with the Q- and N-modules even after the loss of P_P_-module subunits whereas in mammalian CI the P_D_-module is more easily dissociated (Sazanov et al., 2000; Angerer et al., 2011; Letts et al., 2016a). These data indicate that NDUFA11 plays an important role in stabilizing the interaction between the P_D_- and P_P_-modules and that this role has been diminished in mammals. The predominantly lipid-mediated contacts between CI and NDUFA11 in mammals, is also likely the reason why NDUFA11 is easily lost or disordered by detergent when mammalian CI is extracted from the membrane (Letts et al., 2019).

In the mammalian supercomplex I + III_2_ structure, NDUFA11 forms the only interface between CI and CIII in the membrane. The interface is largely formed by interactions between NDUFA11, UQCRB, and UQCRQ (Letts et al., 2019). NDUFA11 therefore plays a role in stabilizing supercomplex I + III_2_ (Letts et al., 2019) and conversely CIII_2_ helps to stabilize NDUFA11 onto the surface of CI (Letts and Sazanov, 2017). The fact that in mammals NDUFA11’s stabilizing role at the interface of the P_P_ and P_D_ modules is diminished relative to *Y. lipolytica*, and that NDUFA11 plays an important role in the formation of supercomplex I + III_2_, leads to the hypothesis that NDUFA11 has been coopted to promote supercomplex formation in mammals. In this scenario CI stability would depend in part on interaction with CIII_2_ through the stabilization of NDUFA11 onto the CI core subunits.

## Conclusion

The functional roles of the multitude of CI accessory subunits have remained mysterious. However, the recent trove of CI structures across bacteria and eukaryotes provides a wealth of information to explore possible functional roles. Comparing the available structures and integrating information from assembly, knockout, knockdown, mutagenesis and clinical studies have allowed us to propose new functional hypotheses for most accessory subunits that can now be experimentally tested. Overall, we hypothesize that CI accessory subunits have roles in 1) coordinating CI assembly, e.g., preventing the association of the N-module until the membrane arm is fully assembled (NDUFS4, NDUFS6, and NDUFA12); 2) providing a scaffold to localize proteins to the inner surface of the cristae (NDUFV3); 3) allowing CI to alter its activity in response to ROS (NDUFA2); 4) integrating information about upstream energy supplies (LYR/ACP pairs) and downstream energy output (NDUFA10); 5) regulating access to the Q-tunnel in an assembly or state dependent manner (NDUFA12, NDUFA9, and NDUFA1); 6) linking CI assembly to cell proliferation (NDUFA13); 7) linking CI assembly mitochondria health (e.g. the CX_9_C motif subunits NDUFA8, NDUFS5, NDUFB10 and NDUFB7, as well as all the TM and matrix subunits that require membrane potential for import into or across the IMM); 8) coupling CI assembly to the redox state of the cell (CX_9_C motif subunits); 9) regulating CI biogenesis via the unfolded protein response (NDUFB2) or alternative splicing (NDUFB11); 10) regulating supercomplex formation (NDUFB3, NDUFB4, NDUFB9, and NDUFA11); and 11) stabilizing the association of lipids at key subunit interfaces (NDUFA3, NDUFC2, NDUFC1, NDUFB1, NDUFB11, NDUFB5, and NDUFB6). We anticipate that this framework for the potential functions of CI’s accessory subunits will contribute to an experimental roadmap to build on the structural, cellular, genetic and biochemical work of the last decades to fully understand the roles of this mysterious multitude in health and disease.
